# Supplementary material for: Facial mimicry interference reduces working memory accuracy for facial emotion expressions
Source: PLoS One. 2024 Jun 26;19(6):e0306113. doi: 10.1371/journal.pone.0306113 (PMC11207140; doi:10.1371/journal.pone.0306113)
Supplement: S1 Appendix — (DOCX) [file pone.0306113.s001.docx]

**S1 Appendix. Post hoc power simulations.**

We followed power simulation guidelines for generalized linear mixed (glmer) models proposed by Kumle et al. [1]. For R script, see: https://osf.io/yhq47/?view_only=9d22e7feb6e043829c1735bc191fbc44

An artificial glmer model was generated in a first step (see Kumle et al., [1]), to specify main effects of working memory load, low interference, and high interference, as well as the interactions between working memory load and each interference condition. Thus, only the experimental effects in our design were included in the model, with no covariates. This means that the output from these simulations cannot be compared to unstandardized effects of covariates (e.g., arousal) reported in the main text, because these are on a different scale than the unstandardized effects of the factors.

Based on the artificial model, we performed a total of nine power simulations using different levels of a Smallest Effect Size of Interest (SESOI) for the interaction between working memory load and high interference. In all models, the main effects and the interaction between working memory load and low interference were set to fixed values (as defined in the artificial model). Across the nine models (including the artificial model generated in the first step), SESOIs for the interaction between working memory load and high interference varied from .10 up to .50, increasing the effect in .05 increments for each new model generated. Power was simulated for all levels of the effect.

When the power simulations were done on each of the nine SESOIs, we extracted power estimates from the simulation outputs. SESOIs and power estimates were fitted onto a power plot to enable identification of level of power for effects in the range of .10-.50 (see below).


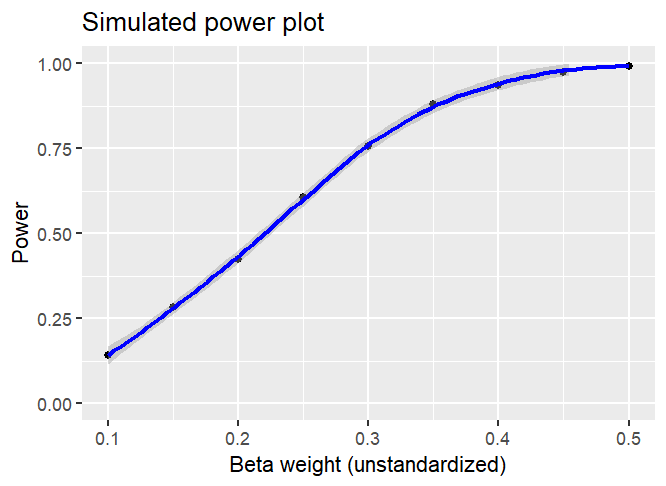


1. Kumle L, Võ MLH, Draschkow D. Estimating power in (generalized) linear mixed models: An open introduction and tutorial in R. Behav Res Methods. 2021;53: 2528–2543. doi:10.3758/s13428-021-01546-0
