# Supplementary material for: Facial mimicry interference reduces working memory accuracy for facial emotion expressions
Source: PLoS One. 2024 Jun 26;19(6):e0306113. doi: 10.1371/journal.pone.0306113 (PMC11207140; doi:10.1371/journal.pone.0306113)
Supplement: Attachment — Submitted filename: PONE-D-23-06734_R1_review.pdf [file pone.0306113.s003.pdf]

In the revised manuscript titled "Facial mimicry interference reduces working memory accuracy for facial emotion expressions when task load is low but not when it is high," the authors investigated the effect of blocking spontaneous facial mimicry on working memory recall for facial emotion expressions. They reported that high-level mimicry interference was effective for 1-back but not for 2-back recalls. The authors made great efforts in addressing comments from the editor and reviewers 1 & 2. However, key points about the study's affordance to provide enough precision for their observed effects were not addressed.

1. The authors stated that they have followed Lakens' suggestions (Lakens, 2022) in sample size justification. While Lakens provided six principles, the authors only stated reasons for resource limitations and heuristics. I want to emphasize that the authors need to demonstrate whether their present study provided precision in effect estimation for what they expected to observe and what they actually observed. The issue of precision in effect estimation is demonstrated in (Lakens & Evers, 2014) Table 1. Small effects require a large sample size to reach the point of stability in effect estimation. With an insufficient sample size, the observed effects are likely unreliable and unlikely to be reproduced. Therefore, I would like to request explicitly:

    a. A sensitivity analysis to determine, at the current design and sample size, what the minimally detectable effect size (MDES) is with 80% power. Note that this has nothing to do with your observed effect size. In Lakens' terms (Lakens, 2022), this is "which minimal effect size will be statistically significant."

    b. Reports of observed effect sizes for all statistical results (Green & MacLeod, 2016; Nakagawa & Schielzeth, 2013).

    c. Considering whether the present study afforded enough precision in effect estimation for the significant and null results they reported in the manuscript. Suppose the effect size reported is much smaller than the present study's precision. In that case, the results reported should not be considered conclusive, and I will have difficulty recommending publishing this study as it is.

2. The discussions focused on the unexpected results and the limitations. While it is okay to speculate that lexico-semantic representations might come into play when working memory demands increase (lines 415-434), the authors should note explicitly that the present study provided no evidence of whether it was the case with their participants.

3. The lack of facial EMG validation of the mimicry interference was apparent. The authors first defended that the placement of electrodes is not compatible with the interference manipulation. Still, they later cited (Davis et al., 2017) who did such validation to strengthen their "assumption" that their manipulation was successful. I suggest it is enough to acknowledge that some validation should have been done, but it is missing in the present study.

4. As mentioned by the editor, participants' facial video recordings might also help validate the manipulation. While the automated FACS (e.g., OpenFace or Py-Feat) might be ineffective in estimating AU12 and AU4 with the plastic foam rob and tapes in place, human FACS raters might be able to tackle this issue. Thus, facial video recordings could be mentioned as a validation method.

Bibliography

Davis, J. D., Winkielman, P., & Coulson, S. (2017). Sensorimotor simulation and emotion processing: Impairing facial action increases semantic retrieval demands. *Cognitive, Affective, & Behavioral Neuroscience*, *17*(3), 652–664. https://doi.org/10.3758/s13415-017-0503-2

Green, P., & MacLeod, C. J. (2016). SIMR: An R package for power analysis of generalized linear mixed models by simulation. *Methods in Ecology and Evolution*, *7*(4), 493–498. https://doi.org/10.1111/2041-210X.12504

Lakens, D. (2022). Sample Size Justification. *Collabra: Psychology*, *8*(1), 33267. https://doi.org/10.1525/collabra.33267

Lakens, D., & Evers, E. R. K. (2014). Sailing From the Seas of Chaos Into the Corridor of Stability: Practical Recommendations to Increase the Informational Value of Studies. *Perspectives on Psychological Science*, *9*(3), 278–292. https://doi.org/10.1177/1745691614528520

Nakagawa, S., & Schielzeth, H. (2013). A general and simple method for obtaining R^2 from generalized linear mixed-effects models. *Methods in Ecology and Evolution*, *4*(2), 133–142. https://doi.org/10.1111/j.2041-210x.2012.00261.x
