# Supplementary material for: Facial mimicry interference reduces working memory accuracy for facial emotion expressions
Source: PLoS One. 2024 Jun 26;19(6):e0306113. doi: 10.1371/journal.pone.0306113 (PMC11207140; doi:10.1371/journal.pone.0306113)
Supplement: Attachment — Submitted filename: PONE-D-23-06734_R2_review.pdf [file pone.0306113.s005.pdf]

Again, the authors made respectable efforts to improve the coherence and transparency of their work. I have only three suggestions.

Major points:

1. Please check whether the r2glmm package (https://cran.r-project.org/web/packages/r2glmm/readme/README.html) (Jaeger et al., 2017) is applicable in providing standardized effect size estimation (semi-partial R^2) for fixed effects in GLMM. If applicable, please add the standardized effect size information for all reported effects accordingly. If not, please provide the rationale, which could be judged by the editor, and I will have no further requests on this issue. The authors' simulation using *simr* was independent of the observed effect size, and I consider this approach valid.

Minor points:

2. Ensuring the R code version on the OSF depository is up to date is crucial for the replicability of your analysis in its latest form. I kindly request you to prioritize this update.
3. Please consider making the review process public during acceptance (I appreciate that PLoS One provides this option). This will provide the reader with the context for why the authors conducted additional analysis or simulation.

Jaeger, B. C., Edwards, L. J., Das, K., & Sen, P. K. (2017). An R^2 statistic for fixed effects in the generalized linear mixed model. *Journal of Applied Statistics*, *44*(6), 1086–1105. https://doi.org/10.1080/02664763.2016.1193725
