# Supplementary material for: Facial mimicry interference reduces working memory accuracy for facial emotion expressions
Source: PLoS One. 2024 Jun 26;19(6):e0306113. doi: 10.1371/journal.pone.0306113 (PMC11207140; doi:10.1371/journal.pone.0306113)
Supplement: Attachment — Submitted filename: Response to reviewers.docx [file pone.0306113.s002.docx]

**Editor comments:**

I want to begin by apologizing for the lengthy delay in getting a recommendation to you. I had sustained difficulty securing reviewers for your submission, with some reviewers failing to submit their report, requiring me to secure new reviewers and further delaying a final recommendation. None of this affected my judgement of your manuscript, which I personally enjoyed reading, it's just an unfortunate pattern editor have been dealing with since COVID.

I was fortunate to secure two expert reviewers with knowledge of the field, each of whom provided insightful and helpful reports. As you will see, both reviewers and I had a positive opinion on the quality and clarity of the manuscript. Overall, the manuscript is well written, appropriately cited, and clearly identifies a gap in the mimicry literature; I personally enjoyed reading your work. However, both reviewers and I took issue with certain aspects of the narrative and analyses.

There was consensus across reviewers, including myself, that facial mimicry was not the observed variable in the study, and that this needs to be made much clearer to the reader. The narrative makes mimicry a central piece of the story, yet mimicry was not measured (with EMG or video motion); rather, it was facial expression recall accuracy that was measured. Like R2, the results of the interaction are interesting, and it would be helpful to contextualize these findings with typical n-back literature findings. R1 makes the insightful comment that the study had no control condition with no-facial stimuli; a discussion here is warranted. Reporting of your mixed model can also be improved for clarity. Finally, interpretation of the null, as indicated by R2 is a serious point for consideration given the lack of a prior sample size estimate. Below you will find comments of the two reviewers, along with own additional suggestions.

**R:** Regarding the delay, we are aware of the issues of finding reviewers, and appreciate your effort in doing this. You also seem to have been successful, the comments from both reviewers were useful. We are very grateful for getting the opportunity to submit a revised version of this manuscript. Also, we should apologize for taking perhaps a bit too much time for our revision. From what we understand, you seem to be interested in what we do here, and we wanted to make sure that we did not miss any part that needed to be changed.

The comments in the previous round of review included several different aspects related to the general narrative of the paper, some selections made in the design and analyses, and our conclusions. Regarding the narrative, we now place the study more clearly in the context of the WM literature, arguing that facial mimicry might contribute to the precision of representations based on a resource model perspective of WM. We have also tried to clarify that we did behavioral manipulations of facial mimicry, and that the outcome we were interested in investigating was WM recall for emotion expressions. These changes have mostly impacted on the introduction and the discussion sections. We have also updated the rationale for the sample size, based on Laken’s terminology, and the description of the analysis, after changing the analysis itself based on the detailed comments below. The most important changes are outlined here:

- The title was changed to “*Facial mimicry interference reduces working memory accuracy for facial emotion expressions when task load is low but not when it is high*” which acknowledges that we actually do see an effect when load is low, and that we did not find evidence of the same effect when working memory load is higher. This is different from stating that facial mimicry does not influence working memory processing.
- In the introduction, we now start from the perspective of WM, and ask if (behavioral) interference of (presumed) facial mimicry will produce a negative effect on WM precision. Our perspective on WM is based on a resource model which emphasizes the interacting effects of storage demands and precisions of representations in WM processing. With our manipulations, we assume that facial mimicry interference will reduce precision (probably through impaired sensorimotor simulation), and the increase in load will lead to increased storage demands. Based on this, the argument is that load and interference will produce interacting negative effects on WM recall. However, since we only see the effect of interference when load is low, we think that our data suggest that facial mimicry influences precision when WM storage demands are not too high. This is also reflected in the discussion of the results, and the conclusions. We think this is in line with what you suggest, but expressed within the WM framework we usually apply.
- In the first paragraph of the Discussion (p. 17), we conclude: “*Working memory load had a strong negative effect on precision, but contrary to what was predicted, an effect of facial mimicry interference was only observed when working memory load was low. Thus, we found partial support for the notion that incongruent sensorimotor feedback impairs working memory for facial emotion expressions [35].*” As in the update title, here we both say that there is an effect of facial mimicry when load is low, and that we did not find evidence of the same effect when working memory load was higher. The same idea is also expressed in the updated conclusions (p. 22): “*Facial mimicry might influence the precision of representations of facial emotion expressions when load on working memory is low but perhaps not when load is high. Thus, sensorimotor feedback represents a useful source of information for the processing of emotion expressions when the conditions allow for it.*”
- We have added a rationale for the sample size based on Laken’s terminology (p. 8): “*The sample size was determined based on resource limitations and heuristics [56]. More specifically, previous studies with similar manipulations and designs showed statistically significant effects with samples in the range of 20 to 50 participants [22,25,29,30,57] and such an approach was also deemed feasible in our study given the available funding and the planned balancing of conditions. Thirty-six participants were recruited, and our expected sample size was reached.*”
- We have updated the description of the analysis with more details (p. 12-14): “*To investigate our main prediction that a negative effect of facial mimicry on emotion expression precision increases with increasing working memory load, we performed a generalized linear mixed effects model with the within-group factors specified as working memory load (two levels: low, 1-back, and high, 2-back) and facial mimicry interference (three levels: no, low, and high), as well as their interaction. The dependent variable was whether the response on a given trial was correct. Control factors included the block order to account for potential habituation and mnemonic effects, as well as individual mean valence and mean arousal estimates for the three types of emotional expressions in the stimuli material (angry, happy, and neutral) to model individual differences along these dimensions. The random effect structure included correlated intercepts and slopes for load at the level of the individual. Analysis was performed in R statistical software [69] using the glmer function from the lme4 package [70] for model estimation. For testing the simple effects of interactions, the emmeans package [71] was used. To deal with the binary outcome variable, a logit-link function was applied. The model was estimated using maximum likelihood estimation with the bobyqa optimizer and 1000 iterations. Satterthwaite approximation of degrees of freedom was applied to test fixed effects. Working memory load was dummy-coded, using one variable with low load as the reference at 0. Facial mimicry interference was also dummy-coded, using two variables, one for low interference and another for high interference, with no interference as the reference at 0 in both. We used the package sjPlot [72] to run comprehensive regression diagnostics for our main model, including assessments for variance, homoscedasticity, normality, random effects, and outliers, which consistently validated the model’s conformity to the assumptions. To test the validity of the emotion expressions in the pictures, mean valence and mean arousal ratings for each of the categories angry, neutral, and happy expressions were compared, in two separate analyses. The random effect structure in these analyses included random intercepts at the level of the individual. A more complex random effect structure created convergence issues. The assumption of normality was violated for the rating data since it showed clustering at several areas of the curve. Hence, a Gaussian Mixture Model (GMM) approach was used for these analyses. This approach was applied by using the package mixtools [73] and the function normalmixEM to generate the best fit. It allowed the model to capture complex patterns in the data (non-linear) and perform with accuracy despite the assumption of normality being violated. The effects of angry and happy emotion expressions were modelled using two dummy-variables with neutral emotion expressions as the reference at 0. The model was estimated using maximum likelihood estimation with the Nelder-Mead optimizer and 1000 iterations. No data was missing, and the significance level was set to α = .05 in all statistical analyses. The data file (.csv) and analysis script (in R) for this study are available on the Open Science Framework (see: https://osf.io/yhq47/?view_only=9d22e7feb6e043829c1735bc191fbc44).*”
- We have changed the main analysis, using dummy-coded variables instead of factor variables, and by adding several covariates. The new results reads (p. 14-15): “*For accuracy, there was a main effect of working memory load (β = -1.30, df = 35, p < .001), which means that the higher the load the lower the performance accuracy (see Table 1). Further, there was a negative main effect of high interference, β = -0.61, df = 35, p = .009, but no main effect of low interference, suggesting that performance accuracy in the high interference condition only was poorer than in the no interference condition. We found positive main effects of valence (β = 0.078, df = 35, p < .001) and arousal (β = 0.26, df =35, p = .003), which means that performance accuracy improved when the mean valence and mean arousal were higher. Finally, the main effect of block order was also positive and statistically significant (β = .076, df = 35, p < .001), therefore, participants improved as they progressed in the task. However, the most important finding of our study was that the interaction between working memory load and high interference was statistically significant (β = 0.30, df = 35, p = .024, displayed in Fig 3), but the interaction between working memory load and low interference was not (see Table 1). Following up the simple main effects on the statistically significant interaction revealed that when working memory load was high (2-back), the effect of high interference was not significant (β = 0.00, df = 35, p = 1.00). The pattern and direction of the main and interaction effects suggest that there was a negative effect of the high interference condition when working memory load was low, but not when the load was high (see Fig 3).*”

For responses to all specific comments, see details below. We have attached both a clean version, and a version with tracked changes. The tracked-changes version looks terrible (because of the number of changes), but from what we understood, we should include both in the re-submission. Let us know if we need to prepare another version.

**Comments from editor**

When submitting your revision, we need you to address these additional requirements.

1. Please ensure that your manuscript meets PLOS ONE's style requirements, including those for file naming. The PLOS ONE style templates can be found at 
<https://journals.plos.org/plosone/s/file?id=wjVg/PLOSOne_formatting_sample_main_body.pdf> and 
<https://journals.plos.org/plosone/s/file?id=ba62/PLOSOne_formatting_sample_title_authors_affiliations.pdf>

**R:** We think that the previous version had the wrong formatting of the figures and the table, as well as some minor formatting issues in the text. We apologize for this, and in the new version we have changed the formatting to align with the style requirements.

2. We note that Figure 1 includes an image of a participant in the study. 
As per the PLOS ONE policy (<http://journals.plos.org/plosone/s/submission-guidelines#loc-human-subjects-research>) on papers that include identifying, or potentially identifying, information, the individual(s) or parent(s)/guardian(s) must be informed of the terms of the PLOS open-access (CC-BY) license and provide specific permission for publication of these details under the terms of this license. Please download the Consent Form for Publication in a PLOS Journal (<http://journals.plos.org/plosone/s/file?id=8ce6/plos-consent-form-english.pdf>). The signed consent form should not be submitted with the manuscript, but should be securely filed in the individual's case notes. Please amend the methods section and ethics statement of the manuscript to explicitly state that the patient/participant has provided consent for publication: “The individual in this manuscript has given written informed consent (as outlined in PLOS consent form) to publish these case details”.

**R:** This is changed in the Fig 1 legend (p. 10) and added in adjunct to the ethics statement (p. 8).

• Alexthymia is considered a personality trait, not a medical condition or disorder. See Ricciardi et al, 2015 (Neuropsychiatry) for reference material, or you can just drop the reference to medical condition.

**R:** Thanks for pointing this out. It now reads “*which is the impaired ability to represent, recognize, and verbally label emotional states*” (p. 4).

• Your manuscript is missing a strong justification for sample size. I won’t ask for a posthoc sample size estimate, as this is meaningless. Rather, see Lakens, 2022 for sample size/power justification and follow their advice that matches your situation. In future, please consider the use of simR as a suitable sample size estimate in mixed models. https://humburg.github.io/Power-Analysis/simr_power_analysis.html

**R:** Thanks for the suggestion on using simR for future studies. We have read the Lakens (2022) paper, and the situation best describing the justification for our sample size is resource limitations combined with heuristics. Data collection was based on what we saw as a reasonable number of participants in relation to previous studies (reporting significant results) and the available funding. We also naïvely assumed that the effects would probably be large, since that is usually what we observe in the working memory studies we do (manipulations with effects around d = 1, or stronger, are common). We have updated the justification for the sample size, using Lakens (2022) as a reference (see p. 8):

“*The sample size was determined based on resource limitations and heuristics [56]. More specifically, previous studies with similar manipulations and designs showed statistically significant effects with samples in the range of 20 to 50 participants [22,25,29,30,57] and such an approach was also deemed feasible in our study given the available funding and the planned balancing of conditions. Thirty-six participants were recruited, and our expected sample size was reached.*”

• Your mixed model reporting is missing many key details. Did you investigate the assumptions of mixed models? See Harrison et al, 2018. Normality of residuals, homogeneity of variances etc? sjPlot provides straightforward visual diagnostics.

**R:** Thanks for pointing this out. We now comment of the assumption checks of the model(s) in the paper. All assumptions were tested using the sjPlot package, and this is described in the paper (see p. 13):

“*We used the package sjPlot [72] to run comprehensive regression diagnostics for our main model, including assessments for variance, homoscedasticity, normality, random effects, and outliers, which consistently validated the model’s conformity to the assumptions.*”

• What form of p-value correction are you reporting, Satterthwaite or Kenward-Roger? Luke 2017 suggests that KR is the gold standard. Either way, please include in your manuscript.

**R:** We now report that the Satterthwaite correction was used (see p. 13):

“*Satterthwaite approximation of degrees of freedom was applied to test fixed effects.*”

• What was your random effects structure? I note in your script that with (1|fp), you’re not exploring by-participant random slopes for load or interference; yet a maximal RE structure is recommended to avoid Type I errors; Barr et al, 2013.

**R:** In the revised version, we include random slopes for load in addition to the random intercept, as well as the correlation between these effects. We have specified this in the description of the model (p. 12-13):

“*The random effect structure included correlated intercepts and slopes for load at the level of the individual.*”

• Ideally, you would report other aspects of your model (optimiser used, number of iterations). Specify if you optimised with REML or ML.

**R:** Thanks for pointing this out. We have updated the description of the statistical analysis, now including the requested information (p. 13):

“*The model was estimated using maximum likelihood estimation with the bobyqa optimizer and 1000 iterations*”

• Please specify in the manuscript the distribution used (logit)

**R:** This information is added in the updated description of the statistical analysis (p. 13):

“*To deal with the binary outcome variable, a logit-link function was applied.*”

• I agree with R1’s concerns regarding interpretation of the interaction. You may be better positioned to code the contrasts yourself. You may find this tutorial helpful – <https://marissabarlaz>.github.io/portfolio/contrastcoding/. Optionally, you may want to consider use of the emmeans package and performing pairwise comparisons.

**R:** This was a very helpful suggestion, thanks. We have updated the coding of the conditions as suggested by you and R1 (dummy-coded levels with low load and no interference as the baseline conditions), and we think that this makes the results easier to interpret. For the follow-up of the significant interaction between the new dummy-coded High interference and Load variables, we used emmeans to estimate simple main effects. New results on p. 14-15 in the ms:

“*For accuracy, there was a main effect of working memory load (β = -1.30, df = 35, p < .001), which means that the higher the load the lower the performance accuracy (see Table 1). Further, there was a negative main effect of high interference, β = -0.61, df = 35, p = .009, but no main effect of low interference, suggesting that performance accuracy in the high interference condition only was poorer than in the no interference condition. We found positive main effects of valence (β = 0.078, df = 35, p < .001) and arousal (β = 0.26, df =35, p = .003), which means that performance accuracy improved when the mean valence and mean arousal were higher. Finally, the main effect of block order was also positive and statistically significant (β = .076, df = 35, p < .001), therefore, participants improved as they progressed in the task. However, the most important finding of our study was that the interaction between working memory load and high interference was statistically significant (β = 0.30, df = 35, p = .024, displayed in Fig 3), but the interaction between working memory load and low interference was not (see Table 1). Following up the simple main effects on the statistically significant interaction revealed that when working memory load was high (2-back), the effect of high interference was not significant (β = 0.00, df = 35, p = 1.00). The pattern and direction of the main and interaction effects suggest that there was a negative effect of the high interference condition when working memory load was low, but not when the load was high (see Fig 3).* ”

• Fig 2 axes need better labelling. It is not clear from the Y-axis or caption what your DV is. Please state it clearly (raw accuracy, I believe, from the letter matching n-back task).

**R:** We have revised our results figures (for the main analysis, Fig 3 in the updated ms) to amend with the uncertainties regarding what they display. See p. 16 in the ms, and below:

Fig 3. Interaction plot for Load and High interference.

The estimated probability of a correct responses (y-axis) for the high (turquoise graph) and no (red graph) interference conditions at low and high levels of working memory load (x-axis), based on the linear mixed effects model. The error bars indicate the 95% confidence intervals of the estimates.

• Rating of Radbound images was a good addition, though not really needed given they are validated stimuli. I am curious why you did not tie your AV data to your accuracy data, as also suggested by R2. That is, did you consider adding A/V as continuous predictors in your glmer model?

**R:** We feel happy with that the validation of the images in this new population do not deviate from the original validation. On the suggestion to tie the ratings to the accuracy data, this was not something we considered in the design, but we do think it is a good suggestion. For the updated analysis, we treat these ratings as individually based positions on a type of internal valence and arousal scales. We have added these continuous predictors to the model and see that they both show a positive effect on accuracy (as reported in the copied text in response to one of your previous comments). However, adding these as covariates (and other control variables, as suggested by revs 1-2) did not change the previously observed main and interaction effects. See updated results on p. 14-16, and in Table 1 inserted below:

|  | **Correct response** | | |
| --- | --- | --- | --- |
| *Predictors* | *Odds Ratios* | *CI* | *p* |
| (Intercept) | 31.00 | 18.04 – 53.28 | <0.001 |
| Load | 0.27 | 0.21 – 0.35 | <0.001 |
| High Interference | 0.55 | 0.35 – 0.86 | 0.009 |
| Low Interference | 0.94 | 0.59 – 1.52 | 0.812 |
| Valence | 1.08 | 1.06 – 1.11 | <0.001 |
| Block order | 1.08 | 1.05 – 1.11 | <0.001 |
| Arousal | 1.30 | 1.09 – 1.54 | 0.003 |
| Load * High interference | 1.35 | 1.04 – 1.76 | 0.024 |
| Load * Low interference | 1.02 | 0.77 – 1.33 | 0.912 |
| **Random Effects** | | | |
| σ^2^ | 3.29 | | |
| τ_00 participants_ | 1.34 | | |
| τ_11 participants\|Load_ | 0.20 | | |
| ρ_01 participants_ | -0.85 | | |
| ICC | 0.13 | | |
| N _participants_ | 36 | | |
| Observations | 11664 | | |
| Marginal R^2^ / Conditional R^2^ | 0.10 / 0.22 | | |

**Comments from Reviewer #1:**

This experiment examines the role of facial mimicry in working memory for images of facial expressions. Overall, the methods are nice and straightforward, although the lack of non-face stimuli is a definite limitation. I have a few suggestions for improving the regression model specification and interpretation. I also find myself disagreeing about the authors’ takeaway, that “Facial mimicry does not play a critical role in working memory for emotion expressions” (p. 21; see my point #6).

**R:** Thanks for your kind words on the methods. We agree that a non-face stimuli condition might have improved the design and think this is something to follow up in the next study. you for pointing this out. We also appreciate your suggestions on how to improve model specification. Regarding the conclusion, we realize that it could be more nuanced than in the previous version, which we have tried to keep in mind when revising the paper (see details in responses below). We hope that the substantial changes that we have made to the paper are in line with your expectations.

METHOD

1. A non-trivial limitation of the study design is a lack of non-face stimuli. We cannot know whether the effects of facial mimicry interference are specific to facial expression working memory, or whether the manipulation was just generally distracting and would have impacted accuracy for any type of stimuli. This needs to be addressed in the Discussion.

**R:** Thank you for pointing this out. In the revised version of the ms, we discuss this as a limitation (p. 22):

“*A final and potentially major limitation was the lack of non-face control stimuli. With this control, we could have tested whether the interference effect that we observed for the low-load condition is, as would be expected, specific for facial input. At the same time, it was only the high interference and not the low interference condition that impaired precision, which speaks for that the observed pattern of results was not driven by a general interference effect. However, had the design included a control condition where the task was to store non-face stimuli, we would feel more confident with our conclusion.*”

ANALYSES AND RESULTS

2. I think the dependent variable in the regression is better described as “whether the response on a given trial was correct or incorrect” or something like that to make it explicit that it’s at the trial level and dichotomous. The current phrasing, “the probability of a correct response as the dependent variable” (p. 11), makes it sound like you’ve converted raw trial-level data into percentages.

**R:** The description of the dependent variable has been updated as suggested (p. 12):

“*The dependent variable was whether the response on a given trial was correct.*”

3. The Load variable is a numeric variable coded as 1 and 2—with the interaction term, this means you’re interpreting the main effects for the Interference dummy variables at a nonexistent level of Load, 0 (0-back trial?). If you relevel Load so 1-back is 0 and 2-back is 1, then you can interpret your Interference main effects as being the effect of facial mimicry interference for the 1-back trials. Here’s a blog post about how changing what 0 means for one variable changes the interpretation of another variable when there’s an interaction term: <https://www.r-bloggers.com/2018/05/regression-with-interaction-terms-how-centering-predictors-influences-main-effects/>

**R:** The default when predictors are defined as a factor with two levels in R (which was the case here) is that the lowest value is recoded to 0 and the higher to 1 in the analysis. Thus, changing the coding to 0 and 1 will not change the results. However, we still did the recoding so that the shared data can be used for replicating the analysis in a software that does not have this default setting or does not treat the variable as a factor.

4. It’s really tricky to describe interactions with dummy-coded variables, and I’m not sure that this sentence (p. 12) is complete: “The most important finding of our study was an interaction between working memory load and low interference (β = -.266, df = 34, p < .044) and load with no interference (β = -.270, df =34, p < .042).” It’s really 1) the interaction between Load and the dummy variable comparing low and high interference and 2) the interaction between Load and the dummy variable comparing no interference and high interference. Note that the two interference variables are using “high interference” as the reference level and comparing “low” and “none” to it. Be similarly careful with your wording when explaining the main effects for interference.

**R:** Thank you for pointing this out. We have updated the analysis with new dummy-coded variables (one for high interference, and another for low interference, with no interference as the baseline/reference level), as suggested by the editor. We think this makes the interpretation more straightforward. We have also tried to improve the interpretations in the text, mostly by clarifying the context of them (what each comparison is). See p. 14-15:

**“***For accuracy, there was a main effect of working memory load (β = -1.30, df = 35, p < .001), which means that the higher the load the lower the performance accuracy (see Table 1). Further, there was a negative main effect of high interference, β = -0.61, df = 35, p = .009, but no main effect of low interference, suggesting that performance accuracy in the high interference condition only was poorer than in the no interference condition. We found positive main effects of valence (β = 0.078, df = 35, p < .001) and arousal (β = 0.26, df =35, p = .003), which means that performance accuracy improved when the mean valence and mean arousal were higher. Finally, the main effect of block order was also positive and statistically significant (β = .076, df = 35, p < .001), therefore, participants improved as they progressed in the task. However, the most important finding of our study was that the interaction between working memory load and high interference was statistically significant (β = 0.30, df = 35, p = .024, displayed in Fig 3), but the interaction between working memory load and low interference was not (see Table 1). Following up the simple main effects on the statistically significant interaction revealed that when working memory load was high (2-back), the effect of high interference was not significant (β = 0.00, df = 35, p = 1.00). The pattern and direction of the main and interaction effects suggest that there was a negative effect of the high interference condition when working memory load was low, but not when the load was high (see Fig 3).*“

5. The labels in Figure 4 are unclear to me. Based on the in-text description, you’ve transformed the model coefficients into probabilities (which I tried doing myself and got different values, so maybe showing the formula would help). I would change the labels. For instance, make it clearer that the probability for “Load” is how much the probability of a correct response changes from low to high load. And the “No” probability is how much the probability of a correct response changes from the “High interference” to the “no interference” conditions. Honestly, I’m not sure if that figure is even worth using since it’s pretty confusing and somewhat redundant with Figure 3. Do we really care about the relative effect sizes of these different variables?

**R:** We agree that Fig 4 in the previous version of the paper was redundant, and we have dropped it in the revised version.

DISCUSSION

6. It’s interesting that you found facial mimicry impairment reduced accuracy on the 1-back trials but not the 2-back trials. One possible interpretation is facial mimicry helps you keep the most recent face in working memory, but the 2-back face’s associated simulation is overridden by the 1-back face. It’s hard to imagine how sensorimotor activity could maintain representations of multiple facial expressions at once when they involve opposing facial actions. This interpretation does not lead me to the conclusion that you “did not find convincing support for the notion incongruent sensorimotor feedback impairs working memory for facial emotion expressions although it might impair precision in the absence of a high working memory load.” (p. 15). As an analogy, let’s say I’m trying to keep a melodic phrase in my working memory by repeating it in my head (which recruits auditory system). If I am unable to do it for two melodies simultaneously, you wouldn’t then conclude that the auditory system wasn’t playing a role—you’d conclude that my auditory working memory can only handle 1 melody at a time. I’m happy to be convinced otherwise if the authors have already ruled out this interpretation for themselves.

**R:** We realize that the intended message was not that well represented in the previous version of the ms, for which we apologize. Hopefully, by clarifying in the introduction what the manipulations represent in relation to our perspective on working memory, rephrasing our interpretations of the results, adjusting the title and the general conclusion, the text is now a better representation of our intended story. Here is a summary of the most important changes:

- The title was changed to “*Facial mimicry interference reduces working memory accuracy for facial emotion expressions when task load is low but not when it is high*” which acknowledges that we actually do see an effect when load is low (or, as you say, when you only need to keep the most recent facial expression in memory), and that we did not find evidence of the same effect when working memory load is higher.
- In the introduction, we now start from the perspective of WM, and ask if (behavioral) interference of (presumed) facial mimicry will produce a negative effect on WM precision. Our perspective on WM is based on a resource model which emphasizes the interacting effects of storage demands and precisions of representations in WM processing. With our manipulations, we assume that facial mimicry interference will reduce precision (probably through impaired sensorimotor simulation), and the increase in load will lead to increased storage demands. Based on this, the argument is that load and interference will produce interacting negative effects on WM recall. However, since we only see the effect of interference when load is low, we think that our data suggest that facial mimicry influences precision when WM storage demands are not too high. This is also reflected in the discussion of the results, and the conclusions. We think this is in line with what you suggest, but expressed within the WM framework we usually apply.
- In the first paragraph of the Discussion (p. 17), we conclude: “*Working memory load had a strong negative effect on precision, but contrary to what was predicted, an effect of facial mimicry interference was only observed when working memory load was low. Thus, we found partial support for the notion that incongruent sensorimotor feedback impairs working memory for facial emotion expressions [35].*” As in the update title, here we both say that there is an effect of facial mimicry when load is low (or, as you say, when you only need to keep the most recent facial expression in memory), and that we did not find evidence of the same effect when working memory load was higher. The same idea is also expressed in the updated conclusions (p. 22): “*Facial mimicry might influence the precision of representations of facial emotion expressions when load on working memory is low but perhaps not when load is high. Thus, sensorimotor feedback represents a useful source of information for the processing of emotion expressions when the conditions allow for it.*”

7. I’m confused by this interpretation in the discussion section (p. 17): “working memory demands increase the neurocognitive system responds by filtering out potentially distracting information, as proposed in the task-engagement/distraction trade-off (TEDTOFF) model.” How is sensorimotor activity distracting information? Isn’t it (theoretically) aiding task performance? Distracting externally-generated auditory input in the Sörqvist et al. study is different from internally-generated and task-relevant facial mimicry.

**R:** We have tried to rephrase this to clarify that although the Sörqvist et al. study investigated external interference the same mechanism might apply for internal interference. In relation to this, we have clarified that we believe that the high interference condition, represents incongruent/interfering information that the system needs to deal with. The logic is that when working memory load is high, this specific type of incongruent information does not seem to impact on performance in our study, and, thus, the focal task might force participants to down-prioritize that information (which might be represented in the suppression of amygdala activity reported by Sörqvist et al). We acknowledge that this is a speculative suggestion, and only suggest this as a tentative mechanism (see p .18-19):

“*We further cautiously propose that a general principle of the neurocognitive system is that when working memory demands increase, the system responds by filtering out potentially distracting information. The ability to suppress external stimuli - like background noise - is working memory dependent. Especially in high load or highly distracting conditions. This is well articulated in the task-engagement/distraction trade-off (TEDTOFF) model [77] and shown across different populations. Because the TEDTOFF model was primarily conceived and developed for audio-verbal stimuli, we only tentatively suggest that suppression and focal engagement of working memory resources in high-load conditions also apply to facial mimicry. However, we assume that the high interference condition produced incongruent information about the stimuli, and consequently, reduced the precision of the representation of the emotional expressions (i.e., internal interference). Thus, when processing becomes more difficult, the focal task might shield out potential distractors, and this could apply both when interference is external and when it is internal.* ”

8. The discussion is a tad repetitive. For instance, the paragraph starting on p. 19 with the phrase “In addition to the potential role of mimicry” was a restatement of earlier ideas.

**R:** We have deleted this paragraph and integrated the most relevant parts of it in other paragraphs of the Discussion. We have also removed some other segments of the Discussion that were only loosely related to the main argument.

9. “…and the sample size was too small to test a three-way interaction” (p. 21). Is this true, given that the additional variable is also within-subject?

**R:** Since we did not do a proper power analysis prior to the experiment, we honestly do not know. Because of this, we also deleted this comment in the discussion. Still, it is true that we did not design the study to test a three-way interaction (but a working memory task with no variability in the stimuli to be remembered is not really a very good working memory task). On the suggestion from the editor, we have added the valence and arousal ratings that participants did as covariates. We believe that these ratings serve as an index of individual differences in how the stimuli was perceived, and controls for this variability when assessing the effects of load and mimicry interference. When adding the covariates to the model, we see that they have a positive effect on performance, but also that the previously reported main and interaction effects remain. The updated results include these covariates, as well as block order (see p. 15):

|  | **Correct response** | | |
| --- | --- | --- | --- |
| *Predictors* | *Odds Ratios* | *CI* | *p* |
| (Intercept) | 31.00 | 18.04 – 53.28 | <0.001 |
| Load | 0.27 | 0.21 – 0.35 | <0.001 |
| High Interference | 0.55 | 0.35 – 0.86 | 0.009 |
| Low Interference | 0.94 | 0.59 – 1.52 | 0.812 |
| Valence | 1.08 | 1.06 – 1.11 | <0.001 |
| Block order | 1.08 | 1.05 – 1.11 | <0.001 |
| Arousal | 1.30 | 1.09 – 1.54 | 0.003 |
| Load * High interference | 1.35 | 1.04 – 1.76 | 0.024 |
| Load * Low interference | 1.02 | 0.77 – 1.33 | 0.912 |
| **Random Effects** | | | |
| σ^2^ | 3.29 | | |
| τ_00 participants_ | 1.34 | | |
| τ_11 participants\|Load_ | 0.20 | | |
| ρ_01 participants_ | -0.85 | | |
| ICC | 0.13 | | |
| N _participants_ | 36 | | |
| Observations | 11664 | | |
| Marginal R^2^ / Conditional R^2^ | 0.10 / 0.22 | | |

GENERAL

10. The paper needs to be proofread, as I encountered the occasional typo or grammar issue throughout. For instance, this sentence on p. 18 is not grammatically correct and is difficult to follow: "Blocker and McIntosh finding of suppressed facial mimicry responses when working memory load increases, speaks for that facial mimicry might not play a crucial role in accurately representing facial emotion expressions when working memory load is high.” Possible rewording: “Blocker and McIntosh’s finding, that increases in working memory load suppress facial mimicry, suggests that facial mimicry might not play a crucial role in accurately representing facial emotion expressions when working memory load is high.”

**R:** We have changed this sentence. The revised ms has also been language-edited by a fluent English-speaking anonymous reviewer.

**Comments from Reviewer #2:**

In this manuscript, the authors systematically address a gap in the literature regarding the effects of interfering with facial mimicry of emotions on working-memory performance at different loads. For this purpose, they assessed 36 students in their 20s (72% female) on a 2 x 3 within-subjects design with two levels of working memory and three levels of interference with facial mimicry. The main DV was accuracy on an emotional n-back task. The Introduction is well-written, presenting relevant background to support a clear rationale. The authors expected a cumulative effect of the 2 factors on accuracy, but the observed interaction was such that an interference effect was only significant in the low working-memory load condition, where low and no interference appeared similarly superior to high interference. A main effect of load was also observed. These effects are generally consistent with prior studies. What is most novel is the lack of interference effects in the high working-memory load condition. Although EMG and subjective reports were not included to verify the interference manipulations, the authors adequately address these limitations. Overall, they conclude that facial mimicry does not play a critical role in working memory for emotional expressions, but may influence performance when working-memory demands are low. However, the interpretive claims rest on the null finding at high load, which deserves further attention, along with other aspects regarding the design and analyses.

**R:** Thanks for your kind words on our paper. We agree that we accidentally fell into focusing perhaps more on the null-finding than the significant effects in the previous version of the manuscript (probably because it was surprising), which we have tried to adjust in the revised version. Below you will find detailed responses to each of your concerns. We hope that the new version of our paper meets your expectations.

MAIN COMMENTS

1. As above, the main conclusion that facial mimicry does not play a critical role in working memory for emotional expressions rests on the failure to observe an effect of interference in the 2-back condition. The Discussion briefly notes that performance was still above chance (p.18) and the figures suggest there was still substantial variation at high load. What was chance? Was the distribution skewed? A counter-argument should be made to the alternative explanation that the absence of effect may be due to a floor effect or restricted range. Moreover, Bayes factors would be helpful towards supporting the strong claims in favour of the null; at present, null hypothesis significant testing cannot support the claims as currently phrased.

**R:** First, we have run the analyses in a bayesian framework (using brms package in R) to quantify Bayes Factors, which did not provide strong evidence for the null. Since we couldn’t find evidence for the null, we have decided not to change our main analysis but toned down the claims and interpretations based on a null-effect. We try to better acknowledge the limitations of the present study in relation to the null-effect on the high level of load in the revised Discussion. For example, on p. 20 we conclude: “*…we did not see that facial mimicry interference influenced precision when working memory load was high. This could be due to the relatively small sample or features of the task design. Thus, the effect of facial mimicry when working memory load is high should be investigated in larger studies using different experimental settings before any firm conclusions can be drawn.*“

Besides acknowledging the limitations of the study, we focus less on the null-result in our general conclusions. The most important changes in our new version of the ms related to this are summarized as:

- The title was changed to “*Facial mimicry interference reduces working memory accuracy for facial emotion expressions when task load is low but not when it is high*” which acknowledges that we actually do see an effect when load is low, and that we did not find evidence of the same effect when working memory load is higher. This is different from stating that there is no effect when working memory load is high.
- In the introduction, we now start from the perspective of WM, and ask if (behavioral) interference of (presumed) facial mimicry will produce a negative effect on WM precision. Our perspective on WM is based on a resource model which emphasizes the interacting effects of storage demands and precisions of representations in WM processing. With our manipulations, we assume that facial mimicry interference will reduce precision (probably through impaired sensorimotor simulation), and the increase in load will lead to increased storage demands. Based on this, the argument is that load and interference will produce interacting negative effects on WM recall. However, since we only see the effect of interference when load is low, we think that our data suggest that facial mimicry influences precision when WM storage demands are not too high. This is also reflected in the discussion of the results, and the conclusions.
- In the first paragraph of the Discussion (p. 17), we conclude: “*Working memory load had a strong negative effect on precision, but contrary to what was predicted, an effect of facial mimicry interference was only observed when working memory load was low. Thus, we found partial support for the notion that incongruent sensorimotor feedback impairs working memory for facial emotion expressions [35].*” As in the update title, here we both say that there is an effect of facial mimicry when load is low, and that we did not find evidence of the same effect when working memory load was higher. The same idea is also expressed in the updated conclusions (p. 22): “*Facial mimicry might influence the precision of representations of facial emotion expressions when load on working memory is low but perhaps not when load is high. Thus, sensorimotor feedback represents a useful source of information for the processing of emotion expressions when the conditions allow for it.*”

Further, the comment on chance-level performance was deleted from the Discussion because it did not add to the argument in the paragraph where it was placed. That paragraph was (and is) about individual variability, and in the revised ms we now focus on what those sources could be by linking our thoughts in the previous version of the ms to some new ideas based on the updated results when adding valence/arousal as predictors in the model (p. 20-21):

“*In one model of working memory for emotions [47–49], inputs are assumed to be evaluated on a positive-negative internal valence scale inducing a feeling state simulation. Although this is related to facial mimicry, as sensorimotor simulation could activate feeling states [35], it is not likely to be determined by facial mimicry alone [39]. Thus, we cannot say that participants did not at all use simulation to solve the task, since simulation of feeling states might be driven by mechanisms not targeted with the type of manipulations applied in the present study [83]. Unfortunately, we did not assess participants’ feeling states during the experiment, nor which strategies they used to solve the task. However, based on the valence and intensity ratings that participants completed, the stimuli seemed to invoke the sought emotional dimensions along the positive-negative continuum. This provides some evidence that the different categories of emotion expressions invoked different feelings in the participants. That the valence and arousal ratings were associated with task performance, further suggests that the perceived emotional content of the stimuli might explain some variability in the precision of working memory representations of emotion expressions. The evidence is somewhat mixed and heterogeneous [84], but some previous studies suggest that the emotional valence of the stimuli might have an impact on working memory performance [85–87], and specifically, working memory for facial emotion expressions [12,13]. In addition, it has been reported that positive emotions are stored with higher precision in working memory than negative emotions [88], a finding that we replicated here. Future studies investigating the effects of facial mimicry and working memory load should consider potential interactions with the perceived valence and arousal dimensions of the stimuli.*”

2. The basis for the sample size should be elaborated or clarified. The methods indicate it was “determined based on previous studies…and thirty-six students participated” (p.7). Does this mean that N=36 was somehow optimal or minimal within a range observed in prior studies? Or did the authors obtain an effect size to calculate and estimated sample size a priori. If so, for what effect(s) of interest? Even if sufficiently powered, it is a relatively small sample size from a reliability / replication standpoint. The Discussion also notes that the “sample size was too small to test a three-way interaction” including valence (p.21). Why wasn’t this considered a priori given the depth of consideration paid to valence in the paper and the field? Additionally, as valence would be embedded within the multilevel model, I’m also not convinced the current design lacks sufficient power.

**R:** We have clarified the motivation for the sample size in the Methods section (p. 8), using Lakens (2022) terminology (available funding and heuristics):

“*The sample size was determined based on resource limitations and heuristics [56]. More specifically, previous studies with similar manipulations and designs showed statistically significant effects with samples in the range of 20 to 50 participants [22,25,29,30,57] and such an approach was also deemed feasible in our study given the available funding and the planned balancing of conditions. Thirty-six participants were recruited, and our expected sample size was reached.* “

Sample size was unfortunately not based on a proper power calculation, since we did not have any data to do such an analysis on. Since we do not know if we had adequate power or not to test the three-way interaction, we deleted that comment from the Discussion. Why valence was not treated as a factor in the design was because mimicry interference (3 levels) and working memory load (2 levels) were the prioritized manipulations based on what we wanted to add to the literature. Already with those two manipulations, we believe that the design was complex enough, considering both the need to control the presentation of the manipulations of interest, and the effort and time put into the experiment by the participants. At the same time, variability in valence is an integrated part of the task when you want to investigate how well participants can store facial emotion expressions in working memory (i.e. you need both targets and foils of similar type), as well as when you want to provoke variability in facial mimicry responses. Thus, we could not use a task that did not include changes in the valence dimension. On the suggestion from the editor, we have added the valence and arousal ratings that participants did as covariates. We believe that these ratings serve as an index of individual differences in how the stimuli was perceived, and controls for this variability when assessing the effects of load and mimicry interference. When adding the covariates to the model, we see that they have a positive effect on performance, but also that the previously reported main and interaction effects remain. The updated results include these covariates, as well as block order (see p. 15):

|  | **Correct response** | | |
| --- | --- | --- | --- |
| *Predictors* | *Odds Ratios* | *CI* | *p* |
| (Intercept) | 31.00 | 18.04 – 53.28 | <0.001 |
| Load | 0.27 | 0.21 – 0.35 | <0.001 |
| High Interference | 0.55 | 0.35 – 0.86 | 0.009 |
| Low Interference | 0.94 | 0.59 – 1.52 | 0.812 |
| Valence | 1.08 | 1.06 – 1.11 | <0.001 |
| Block order | 1.08 | 1.05 – 1.11 | <0.001 |
| Arousal | 1.30 | 1.09 – 1.54 | 0.003 |
| Load * High interference | 1.35 | 1.04 – 1.76 | 0.024 |
| Load * Low interference | 1.02 | 0.77 – 1.33 | 0.912 |
| **Random Effects** | | | |
| σ^2^ | 3.29 | | |
| τ_00 participants_ | 1.34 | | |
| τ_11 participants\|Load_ | 0.20 | | |
| ρ_01 participants_ | -0.85 | | |
| ICC | 0.13 | | |
| N _participants_ | 36 | | |
| Observations | 11664 | | |
| Marginal R^2^ / Conditional R^2^ | 0.10 / 0.22 | | |

3. While the basic script is posted on OSF, the analytic models could be further elaborated. For instance, how were the levels of load and interference modeled and coded (dummy variables)? This is important for interpreting the sign of the regression coefficients. Why did the high interference condition serve as the reference category in these analyses? Why not the no-interference condition to allow for potentially more meaningful assessments of the interactions between low and high interference with load (vs none and low)? Why was a mixed-effect model used to assess n-back performance, but a traditional ANOVA used to assess valence and arousal/intensity ratings?

**R:** Thank you for the suggestion to change the coding of the variables in the main analysis. We have no good answer to why we did not change this before (we just used the default coding for the factor that we used in R), but your suggestion is of course much better. We now use dummy-coded variables for the interference conditions, with the no interference condition as the reference (0). We think this makes the results easier to interpret, as you say in your comment. For the valence and arousal ratings, we have change to the same family of analysis (mixed-effects model) for consistency (although the new model, because of the distribution of ratings, is more complex than needed to show the expected effects, see p. 16).

4. Moreover, a fair bit of attention is devoted to validating the valence/arousal ratings. The Discussion notes the role of context (p.18) and potential moderation by expression type (p.21). As above, why not also include it as a factor of interest in the analyses? This may prove important if variation across expression types are masking an effect of interference when averaged across valence.

**R:** As described in response to one of your earlier comments, the updated analysis includes the average valence and arousal ratings as covariates in the model. As stated above, we do see that these variables are significant predictors and thus explain some variance in the model. However, including them does not change the previously reported main and interaction effects (see above, and p. 14-16 in the ms):

“*For accuracy, there was a main effect of working memory load (β = -1.30, df = 35, p < .001), which means that the higher the load the lower the performance accuracy (see Table 1). Further, there was a negative main effect of high interference, β = -0.61, df = 35, p = .009, but no main effect of low interference, suggesting that performance accuracy in the high interference condition only was poorer than in the no interference condition. We found positive main effects of valence (β = 0.078, df = 35, p < .001) and arousal (β = 0.26, df =35, p = .003), which means that performance accuracy improved when the mean valence and mean arousal were higher. Finally, the main effect of block order was also positive and statistically significant (β = .076, df = 35, p < .001), therefore, participants improved as they progressed in the task. However, the most important finding of our study was that the interaction between working memory load and high interference was statistically significant (β = 0.30, df = 35, p = .024, displayed in Fig 3), but the interaction between working memory load and low interference was not (see Table 1). Following up the simple main effects on the statistically significant interaction revealed that when working memory load was high (2-back), the effect of high interference was not significant (β = 0.00, df = 35, p = 1.00). The pattern and direction of the main and interaction effects suggest that there was a negative effect of the high interference condition when working memory load was low, but not when the load was high (see Fig 3).* ”

5. The figures suggest sizable variation across participants in accuracy, particularly under high load (although it’s not clear what the error bars reflect or the underlying distribution; see below). It may be useful to account for some of this variability in the analysis.

a. For example, although counterbalanced, were there any order effects of the load or interference conditions? The same stimuli repeated across trials. Namely, 9 face identities were each presented with the 3 emotional expressions (27 images total), such that across the 6 blocks, each image was presented twice and each facial identity 6 times. Might this have introduced some habituation or mnemonic interference across trials?

**R:** We do see an effect of block, such that participants improved as they progressed in the experiment. Block number is used as a covariate in the updated analysis (see copied text above, and p. 15 in the ms).

b. Sources of individual differences may also be contributing to error variance. A practice task was used to reduce baseline performance variability. However, it may be useful to harness the verbal working-memory scores (LNS subscale) as covariate if it relates to task performance(?); consider the arguments made for verbal working-memory involvement particularly at high loads. Likewise, what about other participant characteristics? For example, all the stimuli were of women; did participant sex moderate the results?

**R:** We tested LNS as a covariate, but it was not statistically significant (*p* = 0.41). As mentioned before, valence and arousal ratings, representing the participants’ internal representations of the stimuli set, did predict performance, and was kept in the model. Since conditions were not balanced across sex categories, the number of male (n = 9) and female (n = 27) participants were unbalanced and sexes were not matched on any relevant variables (e.g., working memory), we decided not to add this as a predictor to the model (we do not see what we could learn from doing so). However, we do agree that sex might moderate the effects we report here, although we do not believe this is something we can test with the data that we have. This is something to follow-up in future studies.

MINOR POINTS

6. Alexithymia is not typically considered a “medical condition” per se (p.4). Although there are different views on the construct, it can present on a subclinical spectrum and some view it as a personality trait, for example.

**R:** Thanks for pointing this out. It now reads “*which is the impaired ability to represent, recognize, and verbally label emotional states*” (p. 4).

7. Did the data meet the assumptions for the main analyses (i.e., normality, homogeneity of variance, no outliers)? Only the data for the valence/arousal ratings are provided on OSF.

**R:** Thanks for pointing this out. We now comment of the assumption checks of the model(s) in the paper. All assumptions were tested using the sjPlot package, and this is described in the paper (see p. 13):

“*We used the package sjPlot [72] to run comprehensive regression diagnostics for our main model, including assessments for variance, homoscedasticity, normality, random effects, and outliers, which consistently validated the model’s conformity to the assumptions.*”

The data was available on OSF, but in another file than the valence/arousal ratings (the .sav file, Results_long_202210.sav – the previous masterfile was prepared in SPSS by the persons doing the data collection). We have prepared an updated file that includes all data, and that file is shared on OSF: https://osf.io/yhq47/?view_only=9d22e7feb6e043829c1735bc191fbc44

8. Related, while the valence/arousal figures are box plots, the main n-back accuracy findings are displayed as predicted probabilities with some margin of error (bars not defined). The distributions are thus unclear – the authors might consider displaying a dot plot or at least a box plot for these findings as well.

**R:** We have revised our figures, including the figure texts, and hope the explanation for what you see in Fig 3 (the figure for the main analysis) now better meets your expectations. Since we have updated the coding of the effects (dummy coded) in the analysis, the figure for the main analysis (Fig 3) has a different appearance than in the previous version of the manuscript. We still use an interaction plot for the main analysis, because we believe this best match our statistical model. However, to show the individual variability better, we have clarified that what the graph show is the estimated probability of a correct response, for the conditions in the significant interaction (the interaction between dummy coded high interference and working memory load). We also clarified that the 95% confidence intervals refer to the estimates. We’ve included the updated fig and caption below:

Fig 3. Interaction plot for Load and High interference.

The estimated probability of a correct responses (y-axis) for the high (turquoise graph) and no (red graph) interference conditions at low and high levels of working memory load (x-axis), based on the linear mixed effects model. The error bars indicate the 95% confidence intervals of the estimates.

9. I don’t see any captions for the figures in the file for review – are they missing?

**R:** Figure captions are included in the text and not in the figure files, as per the journal instructions. The caption for Fig 1 can be found on p. 10, Fig 2 on p.11-12, Fig 3 on p. 16, and Fig 4 on p. 17. We have also included them below (unformatted), to ease the review process:

Fig 1. Depiction of the facial mimicry interference manipulations.

Graphic example of the high interference (left) and low interference (right) facial mimicry manipulations. The individual in this manuscript has given written informed consent (as outlined in PLOS consent form) to publish these case details.

Fig 2. Illustration of the structure of the n-back experiment.

An overview of how the facial mimicry interference (no, low, and high interference) and working memory load (low, 1-back, and high, 2-back) conditions were distributed across the six experimental blocks, each including 54 trials, for one participant. The order of conditions was balanced across participants.

Fig 3. Interaction plot for Load and High interference.

The estimated probability of a correct responses (y-axis) for the high (turquoise graph) and no (red graph) interference conditions at low and high levels of working memory load (x-axis), based on the linear mixed effects model. The error bars indicate the 95% confidence intervals of the estimates.

Fig 4. Mean arousal and mean valence ratings as a function of emotion expression.

A) The average arousal rating (y-axis) displayed in bar plots for angry (red), neutral (yellow), and happy (green) emotion expressions (x-axis). The error bars indicate the 1.5 interquartile range, and the black dots are the individual means per participant. B) The average valence rating (y-axis) displayed as bar plots for angry (red), neutral (yellow), and happy (green) emotion expressions (x-axis). The error bars indicate the 1.5 interquartile range, and the black dots are the individual means per participant.
