# Supplementary material for: Facial mimicry interference reduces working memory accuracy for facial emotion expressions
Source: PLoS One. 2024 Jun 26;19(6):e0306113. doi: 10.1371/journal.pone.0306113 (PMC11207140; doi:10.1371/journal.pone.0306113)
Supplement: Attachment — Submitted filename: Response to Reviewers.docx [file pone.0306113.s004.docx]

PONE-D-23-06734R1

Facial mimicry interference reduces working memory accuracy for facial emotion expressions when task load is low but not when it is high

PLOS ONE

Dear Dr. Holmer,

Thank you for submitting your manuscript to PLOS ONE. After careful consideration, we feel that it has merit but does not fully meet PLOS ONE’s publication criteria as it currently stands. Therefore, we invite you to submit a revised version of the manuscript that addresses the points raised during the review process.

Thank you for your patience while we collected the reviewer's feedback. As one of the original reviewers was unable to accept the revision task, a third reviewer (R3) was brought on to provide a new review. The original Reviewer 1 was happy with your revisions and is ready to accept the manuscript. However, R3 has asked for revisions. Most of these relate to statistical reporting. While they do not require the collection of new data, they are significant enough to be classified as major revisions. Therefore, I invite you to revise and resubmit the manuscript to address R3's concerns.

Thank you for the opportunity to resubmit another version of the manuscript. We have carefully considered all comments from R3 and tried to do our best to adhere to their requests. However, because of the statistical model we use (generalized linear mixed model), we do not see a straightforward approach to estimate an MDES and effect size metrics for our fixed effects on comparable scales (see discussions on standardized effect size metrics for glmer in e.g. Rights & Sterba, 2019), which means that we do not think we can do exactly what R3 is asking for. As noted by you in the previous round of reviews, power estimation in glmer models should be approached by using simulations (as implemented in e.g. the simr package, Green & MacLeod, 2016), and this should be done prior to data collection. R3 also indicated that this could be a useful tool for us to estimate effect sizes for fixed effects.

Using the simr and mixedpower packages (following Kumle et al.’s, 2021, tutorial), it is possible to estimate power for a range of effects on the same scale (unstandardized) as the output we get from the glmer model we have used in the manuscript. Using this approach to estimate power for a range of effects given the design we have, seems to be a way to get something along the lines of what R3 is asking for. Thus, to test the precision of our design, we implemented Kumle et al.’s method to estimate power for a range of unstandardized effects of the critical interaction term (starting at -.10, and moving down to -.50 in -.05 increments), keeping the random effect structure and the other fixed effects constant (but excluding the covariates, since these were not part of the original design). The simulations included the generation of an artificial glmer model (using the makeGlmer function in the simr package), based on the experimental design our study have (2 levels of load, 3 levels of facial mimicry interference, with 54 observations in each load by interference combination, and 36 participants = 11664 observations) and using our observed fixed effects as a starting point. Then, we simulated the power of the design for the interaction term by varying the size of it in the steps defined above (from -.10, to -.50). In the graph below, we have fitted a power curve based on the output from our simulations (the betas are plotted as absolute values, so that the curve extends to the right instead of to the left). For a beta of .30 (-.30 in the simulation), we see that the power approaches 80% (76% is the estimate from the simulation). This is just a slightly higher (in absolute terms) value than the observed effect of the interaction in the results we get, which we think suggest that the precision of the model fine (note that the size of the effects from the simulations cannot be directly compared to the size of the effects of the covariates in the model reported in the manuscript, since these are on another scale compared to the factors). For now, we do not see a strong motivation for including the (post hoc) power simulations in the manuscript, but if you or R3 find this useful, we can of course reconsider.

We also noted in the comments from R3 that they have the impression that we draw to strong conclusions based on the interaction we see. We thought that we had soften our claims in the previous round of reviews, but it became clear to us when re-reading the manuscript once again that we had not. One obvious example of this, is that we in the reporting of the results did not comment on the unexpected direction of the interaction effect. Instead, we highlighted the interaction as our most important finding. Our prediction, as stated in the introduction, was in the other direction (lines 151-153: “*We predicted that performance accuracy would decrease with increasing working memory load. We expected this effect to be the strongest when facial mimicry interference was high*”). That the direction of the interaction was unexpected is now clarified in the results, the discussion, and the conclusions, and we have de-emphasized the impact of this finding:

- We have changed the title again, now it reads:

“*Facial mimicry interference reduces working memory accuracy for facial emotion expressions when task load is low*” (we have deleted “*...but not when it is high*”)

- The emphasis on the lack of an effect of mimicry interference at high load in the conclusion has been toned down. In the abstract:

“*We conclude that facial mimicry might support working memory for emotion expressions when task load is low, but that the supporting effect possibly is reduced when the task becomes more cognitively challenging.*” (p. 2)

In the first sentence of the Conclusion:

Was before “*Facial mimicry might influence the precision of representations of facial emotion expressions when load on working memory is low but perhaps not when load is high*”, reads now “*Facial mimicry might influence the precision of representations of facial emotion expressions when load on working memory is low*”. (p. 24)

- We emphasize more clearly that the direction of the interaction was unexpected.

In the Abstract:

Was before “…*the high level of mimicry interference impaired accuracy when working memory load was low (1-back) but not when load was high (2-back)*”, now reads “*…the high level of mimicry interference impaired accuracy when working memory load was low (1-back) but, unexpectedly, not when load was high (2-back)*”. (p. 2)

In the Results:

Was before “*However, the most important finding was that the interaction between working memory load and high interference was statistically significant…*”, now reads “*However, the critical test of our study was the interaction between working memory load and high interference. Surprisingly, the statistically significant interaction was in the opposite direction of our prediction…*”. (p. 15)

And in the Discussion:

Was before “*We further cautiously propose that a general principle of the neurocognitive system is that when working memory demands increase, the system responds by filtering out potentially distracting information*”, now reads “*To explain the unexpected finding that working memory load seems to suppress the negative effect of facial mimicry on precision, we cautiously propose that a general principle of the neurocognitive system is that when working memory demands increase, the system responds by filtering out potentially distracting information*” (p. 20)

Was before “*This could be due to the relatively small sample or features of the task design*”, now reads “*It should be acknowledged that this surprising result could be due to some characteristics of the present sample or features of the task design, possibly limiting the precision of the statistical model even though the total number of observations per condition was high (36 participants, and 54 trials per load by mimicry interference condition)*”. (p. 22)

We hope that these changes make it clear that we do not intend to oversell anything here, we are just trying to make sense out of an unexpected, and potentially very interesting, finding.

In addition to the changes described above, we have we have followed R3’s suggested changes in points 2-4.

References

Green, P., & MacLeod, C. J. (2016). SIMR: An R package for power analysis of generalized linear mixed models by simulation. Methods in Ecology and Evolution, 7(4), 493–498. https://doi.org/10.1111/2041-210X.12504

Kumle, L., Võ, M. L. H., & Draschkow, D. (2021). Estimating power in (generalized) linear mixed models: An open introduction and tutorial in R. Behavior Research Methods, 53(6), 2528–2543.

Rights, J. D., & Sterba, S. K. (2019). Quantifying explained variance in multilevel models: An integrative framework for defining R-squared measures. Psychological Methods, 24(3), 309–338. https://doi.org/10.1037/met0000184

Please submit your revised manuscript by Apr 27 2024 11:59PM. If you will need more time than this to complete your revisions, please reply to this message or contact the journal office at [plosone@plos.org](mailto:plosone@plos.org). When you're ready to submit your revision, log on to <https://www.editorialmanager.com/pone/> and select the 'Submissions Needing Revision' folder to locate your manuscript file.

Please include the following items when submitting your revised manuscript:

- A rebuttal letter that responds to each point raised by the academic editor and reviewer(s). You should upload this letter as a separate file labeled 'Response to Reviewers'.
- A marked-up copy of your manuscript that highlights changes made to the original version. You should upload this as a separate file labeled 'Revised Manuscript with Track Changes'.
- An unmarked version of your revised paper without tracked changes. You should upload this as a separate file labeled 'Manuscript'.

If you would like to make changes to your financial disclosure, please include your updated statement in your cover letter. Guidelines for resubmitting your figure files are available below the reviewer comments at the end of this letter.

If applicable, we recommend that you deposit your laboratory protocols in protocols.io to enhance the reproducibility of your results. Protocols.io assigns your protocol its own identifier (DOI) so that it can be cited independently in the future. For instructions see: [https://journals.plos.org/plosone/s/submission-guidelines#loc-laboratory-protocols](https://eur01.safelinks.protection.outlook.com/?url=https%3A%2F%2Fjournals.plos.org%2Fplosone%2Fs%2Fsubmission-guidelines%23loc-laboratory-protocols&data=05%7C02%7Cemil.holmer%40liu.se%7C905e124b6ad34683c02408dc43ad11c5%7C913f18ec7f264c5fa816784fe9a58edd%7C0%7C0%7C638459657930844138%7CUnknown%7CTWFpbGZsb3d8eyJWIjoiMC4wLjAwMDAiLCJQIjoiV2luMzIiLCJBTiI6Ik1haWwiLCJXVCI6Mn0%3D%7C0%7C%7C%7C&sdata=yiA4tXOhnSZX5bCfko9euE%2Bokro8BIbvNw%2BSDfwVWhE%3D&reserved=0). Additionally, PLOS ONE offers an option for publishing peer-reviewed Lab Protocol articles, which describe protocols hosted on protocols.io. Read more information on sharing protocols at [https://plos.org/protocols?utm_medium=editorial-email&utm_source=authorletters&utm_campaign=protocols](https://eur01.safelinks.protection.outlook.com/?url=https%3A%2F%2Fplos.org%2Fprotocols%3Futm_medium%3Deditorial-email%26utm_source%3Dauthorletters%26utm_campaign%3Dprotocols&data=05%7C02%7Cemil.holmer%40liu.se%7C905e124b6ad34683c02408dc43ad11c5%7C913f18ec7f264c5fa816784fe9a58edd%7C0%7C0%7C638459657930854943%7CUnknown%7CTWFpbGZsb3d8eyJWIjoiMC4wLjAwMDAiLCJQIjoiV2luMzIiLCJBTiI6Ik1haWwiLCJXVCI6Mn0%3D%7C0%7C%7C%7C&sdata=0XLv6Hvrhop%2Bf5to3%2Ff25qvIzaNa57GwZIhAUl1aYBk%3D&reserved=0).

We look forward to receiving your revised manuscript.

Kind regards,

Steven R Livingstone
Academic Editor
PLOS ONE

[Note: HTML markup is below. Please do not edit.]

Reviewers' comments:

Reviewer's Responses to Questions

**Comments to the Author**

1. If the authors have adequately addressed your comments raised in a previous round of review and you feel that this manuscript is now acceptable for publication, you may indicate that here to bypass the “Comments to the Author” section, enter your conflict of interest statement in the “Confidential to Editor” section, and submit your "Accept" recommendation.

Reviewer #2: All comments have been addressed

Reviewer #3: (No Response)

2. Is the manuscript technically sound, and do the data support the conclusions?

The manuscript must describe a technically sound piece of scientific research with data that supports the conclusions. Experiments must have been conducted rigorously, with appropriate controls, replication, and sample sizes. The conclusions must be drawn appropriately based on the data presented.

Reviewer #2: Yes

Reviewer #3: Partly

3. Has the statistical analysis been performed appropriately and rigorously?

Reviewer #2: Yes

Reviewer #3: Yes

4. Have the authors made all data underlying the findings in their manuscript fully available?

The [PLOS Data policy](https://eur01.safelinks.protection.outlook.com/?url=http%3A%2F%2Fwww.plosone.org%2Fstatic%2Fpolicies.action%23sharing&data=05%7C02%7Cemil.holmer%40liu.se%7C905e124b6ad34683c02408dc43ad11c5%7C913f18ec7f264c5fa816784fe9a58edd%7C0%7C0%7C638459657930862490%7CUnknown%7CTWFpbGZsb3d8eyJWIjoiMC4wLjAwMDAiLCJQIjoiV2luMzIiLCJBTiI6Ik1haWwiLCJXVCI6Mn0%3D%7C0%7C%7C%7C&sdata=v9AJtorCrvI30kvu7vlGj9Of4JAI0jY09ji2CI42B3c%3D&reserved=0) requires authors to make all data underlying the findings described in their manuscript fully available without restriction, with rare exception (please refer to the Data Availability Statement in the manuscript PDF file). The data should be provided as part of the manuscript or its supporting information, or deposited to a public repository. For example, in addition to summary statistics, the data points behind means, medians and variance measures should be available. If there are restrictions on publicly sharing data—e.g. participant privacy or use of data from a third party—those must be specified.

Reviewer #2: Yes

Reviewer #3: Yes

5. Is the manuscript presented in an intelligible fashion and written in standard English?

PLOS ONE does not copyedit accepted manuscripts, so the language in submitted articles must be clear, correct, and unambiguous. Any typographical or grammatical errors should be corrected at revision, so please note any specific errors here.

Reviewer #2: Yes

Reviewer #3: Yes

6. Review Comments to the Author

Please use the space provided to explain your answers to the questions above. You may also include additional comments for the author, including concerns about dual publication, research ethics, or publication ethics. (Please upload your review as an attachment if it exceeds 20,000 characters)

Reviewer #2: (No Response)

Reviewer #3: In the revised manuscript titled “Facial mimicry interference reduces working memory accuracy for facial emotion expressions when task load is low but not when it is high,” the authors investigated the effect of blocking spontaneous facial mimicry on working memory recall for facial emotion expressions. They reported that high-level mimicry interference was effective for 1-back but not for 2-back recalls. The authors made great efforts in addressing comments from the editor and reviewers 1 & 2. However, key points about the study’s affordance to provide enough precision for their observed effects were not addressed.

1. The authors stated that they have followed Lakens’ suggestions (Lakens, 2022) in sample size justification. While Lakens provided six principles, the authors only stated reasons for resource limitations and heuristics. I want to emphasize that the authors need to demonstrate whether their present study provided precision in effect estimation for what they expected to observe and what they actually observed. The issue of precision in effect estimation is demonstrated in (Lakens & Evers, 2014) Table 1. Small effects require a large sample size to reach the point of stability in effect estimation. With an insufficient sample size, the observed effects are likely unreliable and unlikely to be reproduced. Therefore, I would like to request explicitly:
a. A sensitivity analysis to determine, at the current design and sample size, what the minimally detectable effect size (MDES) is with 80% power. Note that this has nothing to do with your observed effect size. In Lakens’ terms (Lakens, 2022), this is “which minimal effect size will be statistically significant.”
b. Reports of observed effect sizes for all statistical results (Green & MacLeod, 2016; Nakagawa & Schielzeth, 2013).
c. Considering whether the present study afforded enough precision in effect estimation for the significant and null results they reported in the manuscript. Suppose the effect size reported is much smaller than the present study's precision. In that case, the results reported should not be considered conclusive, and I will have difficulty recommending publishing this study as it is.

- In the previous round of revisions, we tried to make it clear that we do not see our results as conclusive – in fact, we were rather surprised by the direction of the interaction. At the start, we expected that the effect of mimicry would be stronger when load was higher, not that it would be diminished at that level. However, even with the earlier revisions, we see that we still focus too much on the interaction effect, and do not adequately acknowledge that the direction of the effect was unexpected. As you indicate in your comment, we sometimes propose too strong conclusions based on the observed null effect when load is high. Thus, we have revised the manuscript paying special attention to this, with the following outcomes:
  - We have changed the title again, now it reads:

“*Facial mimicry interference reduces working memory accuracy for facial emotion expressions when task load is low*” (we have deleted “*...but not when it is high*”)

- The emphasis on the lack of an effect of mimicry interference at high load in the conclusion has been toned down. In the abstract:

“*We conclude that facial mimicry might support working memory for emotion expressions when task load is low, but that the supporting effect possibly is reduced when the task becomes more cognitively challenging.*” (p. 2)

In the first sentence of the Conclusion:

Was before “*Facial mimicry might influence the precision of representations of facial emotion expressions when load on working memory is low but perhaps not when load is high*”, reads now “*Facial mimicry might influence the precision of representations of facial emotion expressions when load on working memory is low*”. (p. 24)

- We emphasize more clearly that the direction of the interaction was unexpected.

In the Abstract:

Was before “…*the high level of mimicry interference impaired accuracy when working memory load was low (1-back) but not when load was high (2-back)*”, now reads “*…the high level of mimicry interference impaired accuracy when working memory load was low (1-back) but, unexpectedly, not when load was high (2-back)*”. (p. 2)

In the Results:

Was before “*However, the most important finding was that the interaction between working memory load and high interference was statistically significant…*”, now reads “*However, the critical test of our study was the interaction between working memory load and high interference. Surprisingly, the statistically significant interaction was in the opposite direction of our prediction…*”. (p. 15)

And in the Discussion:

Was before “*We further cautiously propose that a general principle of the neurocognitive system is that when working memory demands increase, the system responds by filtering out potentially distracting information*”, now reads “*To explain the unexpected finding that working memory load seems to suppress the negative effect of facial mimicry on precision, we cautiously propose that a general principle of the neurocognitive system is that when working memory demands increase, the system responds by filtering out potentially distracting information*” (p. 20)

Was before “*This could be due to the relatively small sample or features of the task design*”, now reads “*It should be acknowledged that this surprising result could be due to some characteristics of the present sample or features of the task design, possibly limiting the precision of the statistical model even though the total number of observations per condition was high (36 participants, and 54 trials per load by mimicry interference condition)*”. (p. 22)

- In the general case, estimating MDES and compare this estimate to observed effects on some standardized metric is useful to inform us about the precision of a statistical model (Lakens, 2022). However, we are not sure that there is a reliable and meaningful way of calculating standardized (e.g., R2) estimates for fixed effects in generalized linear mixed models that could be compared to an MDES estimate on a standardized metric (see, Right & Sterba, 2019, 2020, for a general overview and discussion on effect size estimates for mixed models). The Nakagawa and Schielzeth (2013) approach, later adapted to also deal with models with random slopes in addition to random intercepts (Johnson, 2014; Nakagawa et al., 2017), has been implemented in some packages in R. The most recent development seems to be the partR2 package (Stoffel et al., 2021). The partR2 seems generally useful when estimating overall marginal and conditional effects of the model (although, this has also been criticized, see e.g. …), and it can be used to partition the relative “importance” of the fixed effects in a model. However, from what we understand, these estimates are not easily compared to any type of standardized estimate. Further, the partR2 package does not deal with fixed effects that are involved in a random effect (i.e., in our model, we have random slopes for Load). In sum, we have not found a method that seems to be generally accepted to be used to do exactly what you are asking for, but if you have a method in mind that can achieve this for a generalized linear mixed models with a random intercept and random slope, we are open to suggestions.

As noted by the editor in the previous round of review, the simr package (Green & MacLeod, 2016) can be used to simulate power for generalized linear mixed models, preferably before the study is conducted. You also proposed we can check this reference for the estimation of effect sizes for fixed effects. Kumle et al. (2021) describes a tutorial for how to use the simr and mixedpower packages to simulate power in generalized linear models. With this approach, it is possible to estimate power for a range of effects on the same scale (unstandardized) as the output we get from the model we have used in the manuscript. Thus, to test the precision of our design, we implemented Kumle et al.’s method to estimate power for a range of unstandardized effects of the interaction term (starting at -.10, and moving down to -.50 in -.05 increments), keeping the random effect structure and the other fixed effects constant (but excluding the covariates, since these were not part of the original design). The simulations included the generation of an artificial glmer model (using the makeGlmer function in the simr package), based on the experimental design our study have (2 levels of load, 3 levels of facial mimicry interference, with 54 observations in each load by interference combination, and 36 participants = 11664 observations) and using our observed fixed effects as a starting point. Then, we simulated the power of the design for the interaction term, varied in the steps as defined above (from -.10, to -.50). In the graph below, we have fitted a power curve based on the output from our simulations (the betas are plotted as absolute values, so that the curve extends to the right instead of to the left). For a beta of .30 (-.30 in the simulation), we see that the power approaches 80% (76% is the estimate from the simulation). This is just a slightly higher (in absolute terms) value than the observed effect of the interaction in the results we get, which we think suggest that the precision of the model fine. Still, if the true effects we are investigating are much smaller, maybe closer to .10, then we see in the graph that the design is under-powered (note that the size of the effects from the simulations cannot be directly compared to the size of the effects of the covariates in the model reported in the manuscript, since these are on another scale compared to the factors). If this is the case, the unexpected direction of the interaction might just represent a random finding (c.f., Lakens & Evers, 2014). We hope that our study can inspire further investigations of the how different mechanisms involved in emotion processing might interact, and to carefully consider how working memory load might enhance or suppress the influence of other mechanisms. Still, we cannot more than agree that it would be unwise for us to draw definitive conclusions, and we hope you agree that we have adequately addressed this concern in our revisions with the changes listed above. We are less convinced that adding some type of MDES and standardized effect size estimates, or the results from our post hoc power simulations, will provide a potential reader of the paper with useful information.

Regarding the presentation of the results, we have corrected the degrees of freedom in the model, which was incorrectly specified by N = 36 and not the total number of observations (36 participants and 324 items = 11 664 observations in total) in the last version of the manuscript.

*References*

Green, P., & MacLeod, C. J. (2016). SIMR: An R package for power analysis of generalized linear mixed models by simulation. Methods in Ecology and Evolution, 7(4), 493–498. https://doi.org/10.1111/2041-210X.12504
Johnson, P. C. (2014). Extension of Nakagawa & Schielzeth’s R2 GLMM to random slopes models. *Methods in Ecology and Evolution*, *5*,944–946. doi:10.1111/2041-210X.12225
Kumle, L., Võ, M. L. H., & Draschkow, D. (2021). Estimating power in (generalized) linear mixed models: An open introduction and tutorial in R. Behavior Research Methods, 53(6), 2528–2543. https://doi.org/10.3758/s13428-021-01546-0
Lakens, D. (2022). Sample Size Justification. Collabra: Psychology, 8(1), 33267. https://doi.org/10.1525/collabra.33267
Lakens, D., & Evers, E. R. K. (2014). Sailing From the Seas of Chaos Into the Corridor of Stability: Practical Recommendations to Increase the Informational Value of Studies. Perspectives on Psychological Science, 9(3), 278–292. https://doi.org/10.1177/1745691614528520
Nakagawa, S., & Schielzeth, H. (2013). A general and simple method for obtaining R 2 from generalized linear mixed-effects models. Methods in Ecology and Evolution, 4(2), 133–142. https://doi.org/10.1111/j.2041-210x.2012.00261.x
Nakagawa, S., Johnson, P. C. D., & Schielzeth, H. (2017). The coefficient of determination R2 and intra-class correlation coefficient from generalized linear mixed-effects models revisited and expanded. *Journal of the Royal Society Interface*, *14*, 20170213. doi:10.1101/095851
Rights, J. D., & Sterba, S. K. (2019). Quantifying explained variance in multilevel models: An integrative framework for defining R-squared measures. *Psychological Methods*, *24*(3), 309–338. https://doi.org/10.1037/met0000184
Rights, J. D., & Sterba, S. K. (2020). New Recommendations on the Use of R-Squared Differences in Multilevel Model Comparisons. *Multivariate Behavioral Research*, *55*(4), 568–599. https://doi.org/10.1080/00273171.2019.1660605
Stoffel, M. A., Nakagawa, S., & Schielzeth, H. (2021). partR2: Partitioning R2 in generalized linear mixed models. *PeerJ*, *9*, 1–17. https://doi.org/10.7717/peerj.11414

2. The discussions focused on the unexpected results and the limitations. While it is okay to speculate that lexico-semantic representations might come into play when working memory demands increase (lines 415-434), the authors should note explicitly that the present study provided no evidence of whether it was the case with their participants.

- We have clarified that this idea was not tested in the present study (new text marked here in yellow):

“*As working memory demands increase, lexico-semantic representations and mechanisms subserving the online use of those representations become critical for the successful understanding of communicative signals [5] and other types of processing are down-prioritized [77,80].* *The present study did not test whether access to semantic labels for facial input determines processing efficiency in demanding settings, and this possibility should be investigated in future studies.*” (p. 21)

3. The lack of facial EMG validation of the mimicry interference was apparent. The authors first defended that the placement of electrodes is not compatible with the interference manipulation. Still, they later cited (Davis et al., 2017) who did such validation to strengthen their “assumption” that their manipulation was successful. I suggest it is enough to acknowledge that some validation should have been done, but it is missing in the present study.

- The text has been revised in line with your suggestion, see p. 22-23, lines 493-512 (edited text in yellow below):

“*Facial mimicry is measured with electromyography (EMG) [37], a method that might not be compatible with most mimicry interference manipulations since the method is based on the placement of electrodes to the face and sensitive to disturbances. The present study did not include a validation of whether the facial mimicry manipulations that we used induced the sought effects on mimicry interference applying EMG measurements. Instead, we assumed that the manipulations were valid based on observed changes in behavioral performance reported in previous studies [31,66]. In addition to EMG, it might be possible to test the validity of facial mimicry manipulations by coding filmed facial emotion expressions when manipulations are active, using the Facial Action Coding System [89]. Whether and how facial mimicry manipulations interfere with mimicry activity should be considered carefully in future studies and validated using either EMG or video coding.”*

4. As mentioned by the editor, participants’ facial video recordings might also help validate the manipulation. While the automated FACS (e.g., OpenFace or Py-Feat) might be ineffective in estimating AU12 and AU4 with the plastic foam rob and tapes in place, human FACS raters might be able to tackle this issue. Thus, facial video recordings could be mentioned as a validation method.

- For some reason we overlooked this suggestion in the previous revision, this is now fixed (in the section inserted just above). Thanks for the reminder.

Bibliography
Davis, J. D., Winkielman, P., & Coulson, S. (2017). Sensorimotor simulation and emotion processing: Impairing facial action increases semantic retrieval demands. Cognitive, Affective, & Behavioral Neuroscience, 17(3), 652–664. <https://doi.org/10.3758/s13415-017-0503-2>
Green, P., & MacLeod, C. J. (2016). SIMR: An R package for power analysis of generalized linear mixed models by simulation. Methods in Ecology and Evolution, 7(4), 493–498. <https://doi.org/10.1111/2041-210X.12504>
Lakens, D. (2022). Sample Size Justification. Collabra: Psychology, 8(1), 33267. <https://doi.org/10.1525/collabra.33267>
Lakens, D., & Evers, E. R. K. (2014). Sailing From the Seas of Chaos Into the Corridor of Stability: Practical Recommendations to Increase the Informational Value of Studies. Perspectives on Psychological Science, 9(3), 278–292. <https://doi.org/10.1177/1745691614528520>
Nakagawa, S., & Schielzeth, H. (2013). A general and simple method for obtaining R 2 from generalized linear mixed-effects models. Methods in Ecology and Evolution, 4(2), 133–142. <https://doi.org/10.1111/j.2041-210x.2012.00261.x>

7. PLOS authors have the option to publish the peer review history of their article ([what does this mean?](https://eur01.safelinks.protection.outlook.com/?url=https%3A%2F%2Fjournals.plos.org%2Fplosone%2Fs%2Feditorial-and-peer-review-process%23loc-peer-review-history&data=05%7C02%7Cemil.holmer%40liu.se%7C905e124b6ad34683c02408dc43ad11c5%7C913f18ec7f264c5fa816784fe9a58edd%7C0%7C0%7C638459657930868628%7CUnknown%7CTWFpbGZsb3d8eyJWIjoiMC4wLjAwMDAiLCJQIjoiV2luMzIiLCJBTiI6Ik1haWwiLCJXVCI6Mn0%3D%7C0%7C%7C%7C&sdata=UCp8qRuUa1snAR9ymoLoQT0u70IVNcN2MXIAt7QhnYs%3D&reserved=0)). If published, this will include your full peer review and any attached files.


If you choose “no”, your identity will remain anonymous but your review may still be made public.


**Do you want your identity to be public for this peer review?** For information about this choice, including consent withdrawal, please see our [Privacy Policy](https://eur01.safelinks.protection.outlook.com/?url=https%3A%2F%2Fwww.plos.org%2Fprivacy-policy&data=05%7C02%7Cemil.holmer%40liu.se%7C905e124b6ad34683c02408dc43ad11c5%7C913f18ec7f264c5fa816784fe9a58edd%7C0%7C0%7C638459657930874597%7CUnknown%7CTWFpbGZsb3d8eyJWIjoiMC4wLjAwMDAiLCJQIjoiV2luMzIiLCJBTiI6Ik1haWwiLCJXVCI6Mn0%3D%7C0%7C%7C%7C&sdata=KgnnwO0L7zu%2Bsjrsgg8nKsogUFCwHp7B7KRIOSNn%2BlQ%3D&reserved=0).

Reviewer #2: No

Reviewer #3: No

[NOTE: If reviewer comments were submitted as an attachment file, they will be attached to this email and accessible via the submission site. Please log into your account, locate the manuscript record, and check for the action link "View Attachments". If this link does not appear, there are no attachment files.]

While revising your submission, please upload your figure files to the Preflight Analysis and Conversion Engine (PACE) digital diagnostic tool, [https://pacev2.apexcovantage.com/](https://eur01.safelinks.protection.outlook.com/?url=https%3A%2F%2Fpacev2.apexcovantage.com%2F&data=05%7C02%7Cemil.holmer%40liu.se%7C905e124b6ad34683c02408dc43ad11c5%7C913f18ec7f264c5fa816784fe9a58edd%7C0%7C0%7C638459657930880131%7CUnknown%7CTWFpbGZsb3d8eyJWIjoiMC4wLjAwMDAiLCJQIjoiV2luMzIiLCJBTiI6Ik1haWwiLCJXVCI6Mn0%3D%7C0%7C%7C%7C&sdata=jfJZqcZU9z0BpZxKopSQp13CzmqW7vSqrK2M8j3JSiU%3D&reserved=0). PACE helps ensure that figures meet PLOS requirements. To use PACE, you must first register as a user. Registration is free. Then, login and navigate to the UPLOAD tab, where you will find detailed instructions on how to use the tool. If you encounter any issues or have any questions when using PACE, please email PLOS at [figures@plos.org](mailto:figures@plos.org). Please note that Supporting Information files do not need this step.

*In compliance with data protection regulations, you may request that we remove your personal registration details at any time.* [*(Remove my information/details)*](https://www.editorialmanager.com/pone/login.asp?a=r)*. Please contact the publication office if you have any questions.*
