# Supplementary material for: Facial mimicry interference reduces working memory accuracy for facial emotion expressions
Source: PLoS One. 2024 Jun 26;19(6):e0306113. doi: 10.1371/journal.pone.0306113 (PMC11207140; doi:10.1371/journal.pone.0306113)
Supplement: Attachment — Submitted filename: Response to Reviewers.docx [file pone.0306113.s006.docx]

PONE-D-23-06734R2

Facial mimicry interference reduces working memory accuracy for facial emotion expressions

PLOS ONE

Dear Dr. Holmer,

Thank you for submitting your manuscript to PLOS ONE. I have now received reviews from two expert reviewers. As you will see, the reviews are positive, with reviewer 1 opting to accept and reviewer 2 raising only minor concerns.  For context, I was lucky secure Reviewer 2 for your revision. However, as Reviewer 1 needed to decline due to personal issues, a new Reviewer 1 was located.

- Thanks for the opportunity for another resubmission. We are glad to see that the reviewers overall seem happy with the manuscript. To deal with the last few comments, we have done the following:

1) There was a general comment related to “Journal requirements” about the references, and for us to check whether any study we are referring to has been retracted. We checked doi-numbers for the references against Retraction Watch and based on that search, which resulted in 0 references being identified as a retracted study, we conclude that we can keep all references. The reference list was updated with two minor changes, i.e., corrected doi-numbers for two references.
2) In the last round of reviews, one proposed change was for us to add a sensitivity analysis by comparing MDES to some standardized effect size metric for the effects in our glmer model. We concluded that this probably is not possible with available statistical tools. As a response to this, Rev 3 proposed for us to try the r2glmm package to estimate R2-like effect sizes for individual effects. From what we understand this does not offer a viable solution. Firstly, the documentation of this package states that the r2beta function that is used to estimate effect sizes for individual predictors does not work with glmer models from lme4 (see <https://cran.r-project.org/web/packages/r2glmm/r2glmm.pdf>). Secondly, when we run this function on our model we get an error (*In calc_sgv(nblocks = nclusts, blksizes = obsperclust, vmat = SigHat) : Some SGV estimates are non-finite and have been adjusted*) and some spurious results:

```
> r2beta(model = model, method = 'sgv', data = data)
Effect	Rsq	upper.CL	lower.CL
1	Model 	0.049	0.057	0.042
2	Load	0.017	0.022	0.012
5	valence	0.004	0.006	0.002
6	order	0.002	0.004	0.001
7	arousal	0.001	0.003	0.000
3	High	0.001	0.002	0.000
8	Load:High	0.000	0.002	0.000
4	Low	0.000	0.000	0.000
9	Load:Low	0.000	0.000	0.000
```

Thirdly, even if we assume that the output is valid, we do not see how we could estimate a standardized MDES on the same scale as these R2-like estimates, since R2 estimates for linear models are not comparable. Thus, we will not be able to discuss the sensitivity of our model based on this.

In the last round of reviews, we provided a simulation-based power analysis as an alternative approach to assess the sensitivity of the model. The downside with this approach is that it is based on unstandardized estimates that might not be easy to understand outside the context of the study. However, the unstandardized effects can be directly compared to the output we get from our glmer model. We lean towards that this might be the better approach for our study. In the revised version of the manuscript, we have added a short section discussing the results from our simulations in relation to the effects we see in the study, and we also attach a description of the simulations as an Appendix. The new text included at p. 16-17, lines 356-369, reads as follows:

“*Since no proper power analysis was performed prior to the study, a sensitivity analysis based on post hoc power simulations was conducted. Simulations followed the guidelines from Kumle et al. [75], and we only considered the main manipulations of the experiment (working memory load and facial mimicry interference), which means that the simulation results can only be compared to the effects of those factors in the results from the generalized linear mixed effects model reported above. All effects but the interaction between high interference and working memory load (which was the critical test in the design) were set to a fixed value. The overall pattern of the simulation results suggested that beta weights larger than approximately .30 could be detected with at least 80% power, whereas beta weights in a lower range (.10-.20) revealed a power of less than 50% (for more details, see S1 Appendix). Thus, we cannot fully reject the possibility that poor sensitivity of our design might explain why we did not observe an effect of high interference when working memory load was high.*”

Please submit your revised manuscript by Jul 04 2024 11:59PM. If you will need more time than this to complete your revisions, please reply to this message or contact the journal office at [plosone@plos.org](mailto:plosone@plos.org). When you're ready to submit your revision, log on to <https://www.editorialmanager.com/pone/> and select the 'Submissions Needing Revision' folder to locate your manuscript file.

Please include the following items when submitting your revised manuscript:

- A rebuttal letter that responds to each point raised by the academic editor and reviewer(s). You should upload this letter as a separate file labeled 'Response to Reviewers'.
- A marked-up copy of your manuscript that highlights changes made to the original version. You should upload this as a separate file labeled 'Revised Manuscript with Track Changes'.
- An unmarked version of your revised paper without tracked changes. You should upload this as a separate file labeled 'Manuscript'.

If you would like to make changes to your financial disclosure, please include your updated statement in your cover letter. Guidelines for resubmitting your figure files are available below the reviewer comments at the end of this letter.

If applicable, we recommend that you deposit your laboratory protocols in protocols.io to enhance the reproducibility of your results. Protocols.io assigns your protocol its own identifier (DOI) so that it can be cited independently in the future. For instructions see: [https://journals.plos.org/plosone/s/submission-guidelines#loc-laboratory-protocols](https://eur01.safelinks.protection.outlook.com/?url=https%3A%2F%2Fjournals.plos.org%2Fplosone%2Fs%2Fsubmission-guidelines%23loc-laboratory-protocols&data=05%7C02%7Cemil.holmer%40liu.se%7C7bd67385c1d44290848f08dc78b72eaf%7C913f18ec7f264c5fa816784fe9a58edd%7C0%7C0%7C638517975520541089%7CUnknown%7CTWFpbGZsb3d8eyJWIjoiMC4wLjAwMDAiLCJQIjoiV2luMzIiLCJBTiI6Ik1haWwiLCJXVCI6Mn0%3D%7C0%7C%7C%7C&sdata=V2IiUEhUSYhYCu%2Fbq8sUMJ9tk6BFOZytepXP%2BLpsWos%3D&reserved=0). Additionally, PLOS ONE offers an option for publishing peer-reviewed Lab Protocol articles, which describe protocols hosted on protocols.io. Read more information on sharing protocols at [https://plos.org/protocols?utm_medium=editorial-email&utm_source=authorletters&utm_campaign=protocols](https://eur01.safelinks.protection.outlook.com/?url=https%3A%2F%2Fplos.org%2Fprotocols%3Futm_medium%3Deditorial-email%26utm_source%3Dauthorletters%26utm_campaign%3Dprotocols&data=05%7C02%7Cemil.holmer%40liu.se%7C7bd67385c1d44290848f08dc78b72eaf%7C913f18ec7f264c5fa816784fe9a58edd%7C0%7C0%7C638517975520553433%7CUnknown%7CTWFpbGZsb3d8eyJWIjoiMC4wLjAwMDAiLCJQIjoiV2luMzIiLCJBTiI6Ik1haWwiLCJXVCI6Mn0%3D%7C0%7C%7C%7C&sdata=Lxm4Y9dvBEqiPwqfuhXyq9ENtEDeuuHekWTHwO35Awk%3D&reserved=0).

We look forward to receiving your revised manuscript.

Kind regards,

Steven R Livingstone
Academic Editor
PLOS ONE

Journal Requirements:

Please review your reference list to ensure that it is complete and correct. If you have cited papers that have been retracted, please include the rationale for doing so in the manuscript text, or remove these references and replace them with relevant current references. Any changes to the reference list should be mentioned in the rebuttal letter that accompanies your revised manuscript. If you need to cite a retracted article, indicate the article’s retracted status in the References list and also include a citation and full reference for the retraction notice.

-	We have checked all references’ doi-numbers against Retraction Watch, and the results indicate that none of the studies we reference have been retracted. In the previous version, the doi-number for two references was incorrect/missing and has now been corrected/added (Rönnberg et al., 2022 & Dimberg et al., 2000).

[Note: HTML markup is below. Please do not edit.]

Reviewers' comments:

Reviewer's Responses to Questions

**Comments to the Author**

1. If the authors have adequately addressed your comments raised in a previous round of review and you feel that this manuscript is now acceptable for publication, you may indicate that here to bypass the “Comments to the Author” section, enter your conflict of interest statement in the “Confidential to Editor” section, and submit your "Accept" recommendation.

Reviewer #2: All comments have been addressed

Reviewer #3: (No Response)

2. Is the manuscript technically sound, and do the data support the conclusions?

The manuscript must describe a technically sound piece of scientific research with data that supports the conclusions. Experiments must have been conducted rigorously, with appropriate controls, replication, and sample sizes. The conclusions must be drawn appropriately based on the data presented.

Reviewer #2: Yes

Reviewer #3: Yes

3. Has the statistical analysis been performed appropriately and rigorously?

Reviewer #2: Yes

Reviewer #3: Yes

4. Have the authors made all data underlying the findings in their manuscript fully available?

The [PLOS Data policy](https://eur01.safelinks.protection.outlook.com/?url=http%3A%2F%2Fwww.plosone.org%2Fstatic%2Fpolicies.action%23sharing&data=05%7C02%7Cemil.holmer%40liu.se%7C7bd67385c1d44290848f08dc78b72eaf%7C913f18ec7f264c5fa816784fe9a58edd%7C0%7C0%7C638517975520561821%7CUnknown%7CTWFpbGZsb3d8eyJWIjoiMC4wLjAwMDAiLCJQIjoiV2luMzIiLCJBTiI6Ik1haWwiLCJXVCI6Mn0%3D%7C0%7C%7C%7C&sdata=N7eZsjUiIQ%2Fa%2FIfnJGXULBEW6nQROqy4RXRHr%2BNE4QM%3D&reserved=0) requires authors to make all data underlying the findings described in their manuscript fully available without restriction, with rare exception (please refer to the Data Availability Statement in the manuscript PDF file). The data should be provided as part of the manuscript or its supporting information, or deposited to a public repository. For example, in addition to summary statistics, the data points behind means, medians and variance measures should be available. If there are restrictions on publicly sharing data—e.g. participant privacy or use of data from a third party—those must be specified.

Reviewer #2: Yes

Reviewer #3: Yes

5. Is the manuscript presented in an intelligible fashion and written in standard English?

PLOS ONE does not copyedit accepted manuscripts, so the language in submitted articles must be clear, correct, and unambiguous. Any typographical or grammatical errors should be corrected at revision, so please note any specific errors here.

Reviewer #2: Yes

Reviewer #3: Yes

6. Review Comments to the Author

Please use the space provided to explain your answers to the questions above. You may also include additional comments for the author, including concerns about dual publication, research ethics, or publication ethics. (Please upload your review as an attachment if it exceeds 20,000 characters)

Reviewer #2: Overall, the authors have addressed the bulk of comments raised by R3 in the last review and the tempering of conclusions and clarifications improve the manuscript.

One item to note is that R3 requested that the authors further present a sensitivity analysis based on their sample size and discuss their precision in effect estimation. The authors argue that they are unaware of a mechanism to accurately address this request for their analytic model and provide a lengthy consideration of this item in their response letter. To speak to the sensitivity, they present a power curve based on simulations for a similar model (without covariates) in the response, but this not in the manuscript. While this may be harnessed to offer a rough estimate of an MDES, the values are not comparable given the unstandardized betas reported. An estimate and reporting of standardized effects might address this issue. Alternatively, I wonder if providing an estimate based on a more basic model (i.e., ANCOVA vs. a mixed model) could at least provide a conservative estimate (given the MLM here is more powered). Regardless, the authors have addressed practical reasons for their sample size and tempered the strength of their interpretations accordingly.

- Thanks for acknowledging our efforts to deal with the comments in the last round of reviews. Regarding the sensitivity analysis, see our response to Rev 3 below.

As a minor point, in the new sentence on line 410-411, I believe the authors may have meant to say “…to suppress the negative effect of facial mimicry INTERFERENCE on precision.”

- Thanks for noticing this. Corrected in the revised version.

Reviewer #3: Again, the authors made respectable efforts to improve the coherence and transparency of their work. I have only three suggestions.

Major points:
1. Please check whether the r2glmm package (<https://cran.r-project.org/web/packages/r2glmm/readme/README.html>) (Jaeger et al., 2017) is applicable in providing standardized effect size estimation (semi-partial R2) for fixed effects in GLMM. If applicable, please add the standardized effect size information for all reported effects accordingly. If not, please provide the rationale, which could be judged by the editor, and I will have no further requests on this issue. The authors' simulation using simr was independent of the observed effect size, and I consider this approach valid.

- Thanks for your effort in trying to find a viable approach to standardize effect sizes for the effects in our model. From what we understand, using the r2beta function from r2glmm to get R2-like estimates for individual predictors in glmer models is an experimental approach, and for models built with the lm4 package the package documentation says the function currently only can be used with linear mixed models (see, https://cran.r-project.org/web/packages/r2glmm/r2glmm.pdf). Indeed, when running the function on the model, we see some spurious results (tiny estimates for some of the statistically significant effects, see below) and a warning message (*In calc_sgv(nblocks = nclusts, blksizes = obsperclust, vmat = SigHat) : Some SGV estimates are non-finite and have been adjusted*). If we assume that this function provides valid estimates, the alternative interpretation would be that some of the effects we see are indistinguishable from zero. However, this package (and the earlier packages we have tried) does not seem to be a viable solution, especially not if the goal is to compare to MDES based on a more traditional statistical approach (since pseudo-R2 metrics are not comparable to such estimates).

Output from R:

```
 > r2beta(model = model, method = 'sgv', data = d)
Effect	Rsq	upper.CL	lower.CL
1	Model 	0.049	0.057	0.042
2	Load	0.017	0.022	0.012
5	valence	0.004	0.006	0.002
6	order	0.002	0.004	0.001
7	arousal	0.001	0.003	0.000
3	High	0.001	0.002	0.000
8	Load:High	0.000	0.002	0.000
4	Low	0.000	0.000	0.000
9	Load:Low	0.000	0.000	0.000
```

Taken everything discussed in the previous and the current round of reviews into account, leads us to draw the conclusion that the simulation-based approach is preferrable over the other solutions. Thus, we have added a paragraph to discuss the results from the simulations to the paper (p. 16-17, lines 356-369):

“*Since no proper power analysis was performed prior to the study, a sensitivity analysis based on post hoc power simulations was conducted. Simulations followed the guidelines from Kumle et al. [75], and we only considered the main manipulations of the experiment (working memory load and facial mimicry interference), which means that the simulation results can only be compared to the effects of those factors in the results from the generalized linear mixed effects model reported above. All effects but the interaction between high interference and working memory load (which was the critical test in the design) were set to a fixed value. The overall pattern of the simulation results suggested that beta weights larger than approximately .30 could be detected with at least 80% power, whereas beta weights in a lower range (.10-.20) revealed a power of less than 50% (for more details, see S1 Appendix). Thus, we cannot fully reject the possibility that poor sensitivity of our design might explain why we did not observe an effect of high interference when working memory load was high.*”

The details of the simulation are added to a new supplement (S1 Appendix), and the scripts on OSF have been updated to include the simulations as well.

Minor points:
2. Ensuring the R code version on the OSF depository is up to date is crucial for the replicability of your analysis in its latest form. I kindly request you to prioritize this update.

- We apologize that we missed to update the code on OSF when we changed the analyses. We have now ensured that the data and code available on OSF is up to date, including the code for the power simulations.

3. Please consider making the review process public during acceptance (I appreciate that PLoS One provides this option). This will provide the reader with the context for why the authors conducted additional analysis or simulation.

- We are positive to making the review process public, if the paper is accepted.

Jaeger, B. C., Edwards, L. J., Das, K., & Sen, P. K. (2017). An R 2 statistic for fixed effects in the generalized linear mixed model. Journal of Applied Statistics, 44(6), 1086–1105. <https://doi.org/10.1080/02664763.2016.1193725>
